# Supplementary material for: The interactome of CLUH reveals its association to SPAG5 and its co-translational proximity to mitochondrial proteins
Source: BMC Biol. 2022 Jan 10;20:13. doi: 10.1186/s12915-021-01213-y (PMC8744257; doi:10.1186/s12915-021-01213-y)
Supplement: Supplementary file 1 — Additional file 1:. Figure S1. Generation of 3xHA-CLUH expressing cells and proteomic analysis [file 12915_2021_1213_MOESM1_ESM.pdf]

Figure S1

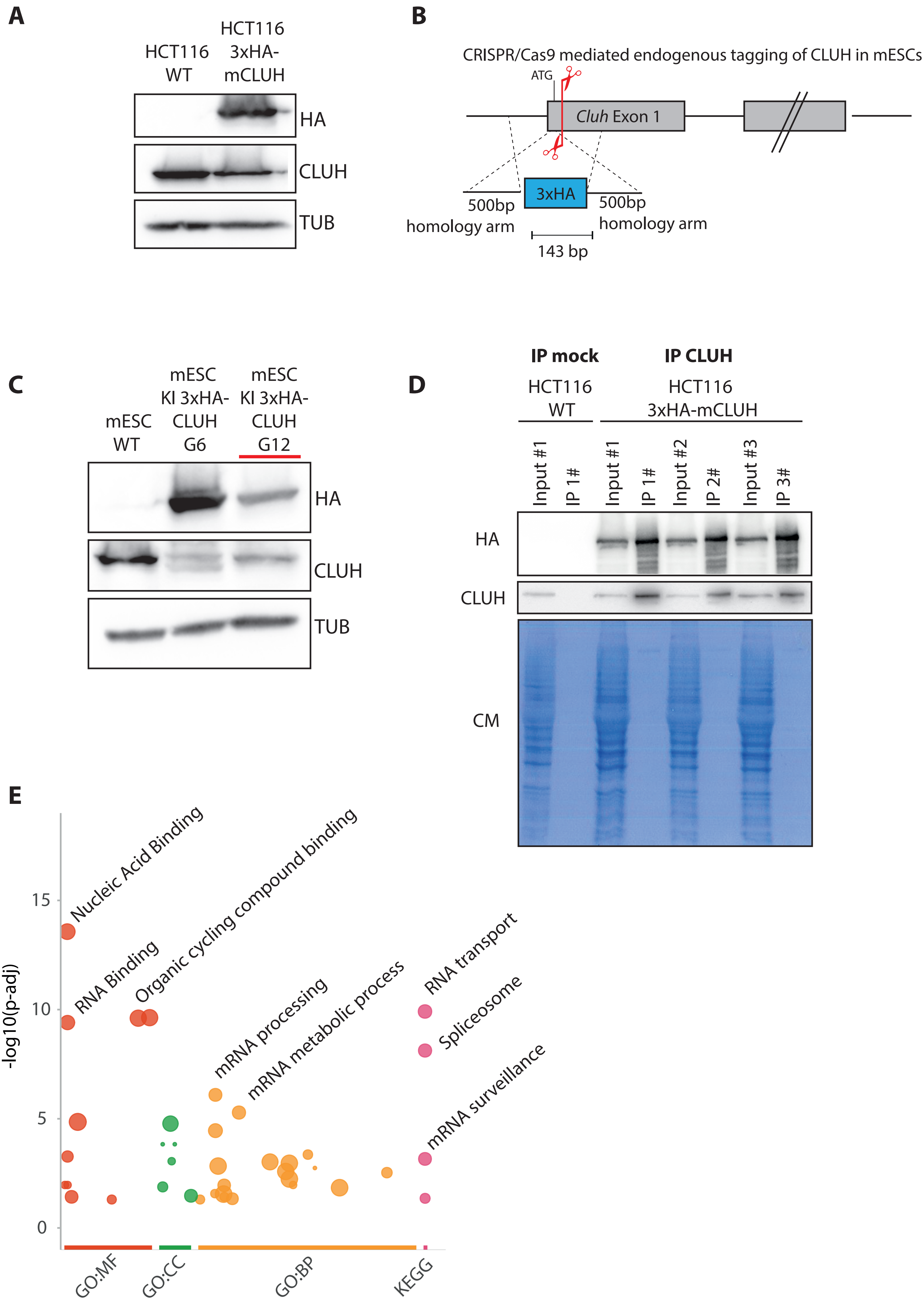

A
HCT116 WT
HCT116 3xHA-mCLUH
HA
CLUH
TUB
B
CRISPR/Cas9 mediated endogenous tagging of CLUH in mESCs
ATG
Cluh Exon 1
3xHA
500bp homology arm
500bp homology arm
143 bp
C
mESC WT
mESC KI 3xHA-CLUH G6
mESC KI 3xHA-CLUH G12
HA
CLUH
TUB
D
IP mock
IP CLUH
HCT116 WT
HCT116 3xHA-mCLUH
Input #1
IP 1#
Input #1
IP 1#
Input #2
IP 2#
Input #3
IP 3#
HA
CLUH
CM
E
-log10(p-adj)
15
10
5
0
Nucleic Acid Binding
RNA Binding
Organic cycling compound binding
mRNA processing
mRNA metabolic process
RNA transport
Spliceosome
mRNA surveillance
GO:MF
GO:CC
GO:BP
KEGG

Figure S1: Generation of 3xHA-CLUH expressing cells and proteomic analysis. (A) Western blot showing the expression of N-terminal HA-tagged mouse CLUH protein (mCLUH) in polyclonal HCT116 stable cell line. Indicated proteins are detected using specific antibodies. **(B)** Schematic representation of the CRISPR/Cas9 mediated knock-in strategy in mESCs, to endogenously tag CLUH in N-terminal with 3xHA. Red scissors indicate the cleavage sites**. (C)** Immunodetection analysis of the endogenously 3xHA-tagged CLUH protein in mESCs. Two selected clones (G6 and G12) are analyzed. The proteins are detected using antibodies directed against CLUH and the HA tag. Coomassie (CM) staining of the membrane is used as loading control. The clone underlined in red was used for the co-IP experiment. **(D)** Western blot analysis of a fraction of total extracts and Co-IP replicate samples sent for LC-MS/MS. Only one representative replicate for the control experiment (HCT116 WT) is shown. The 3xHA-mCLUH protein is detected using anti-HA antibodies, CLUH is detected using specific antibodies and the Coomassie (CM) staining of the membrane is used as loading control. The loaded samples correspond to 1% of the input and 7% of the pulled-down samples. **(E)** Manhattan plot illustrating the gene ontology and pathway enrichment analysis of proteins identified in mESCs CLUH co-IP experiment, generated using G:profiler tool [43]. The functional terms, associated with the protein list, are grouped in four categories: GO: MF (Molecular Function), GO: BP (Biological Process), GO: CC (Cellular Component) and KEGG pathways. The y-axis shows the adjusted enrichment p-values in negative log10 scale. The circle sizes are in accordance with the corresponding term size (in the data base) and terms from the same GO subtree are located close to each other on the x-axis. The most significantly enriched terms are indicated.
