# Supplementary material for: The interactome of CLUH reveals its association to SPAG5 and its co-translational proximity to mitochondrial proteins
Source: BMC Biol. 2022 Jan 10;20:13. doi: 10.1186/s12915-021-01213-y (PMC8744257; doi:10.1186/s12915-021-01213-y)
Supplement: Supplementary file 4 — Additional file 4:. Figure S2. RNA-independent CLUH interaction with SPAG5 and KNSTRN. [file 12915_2021_1213_MOESM4_ESM.pdf]

Figure S2

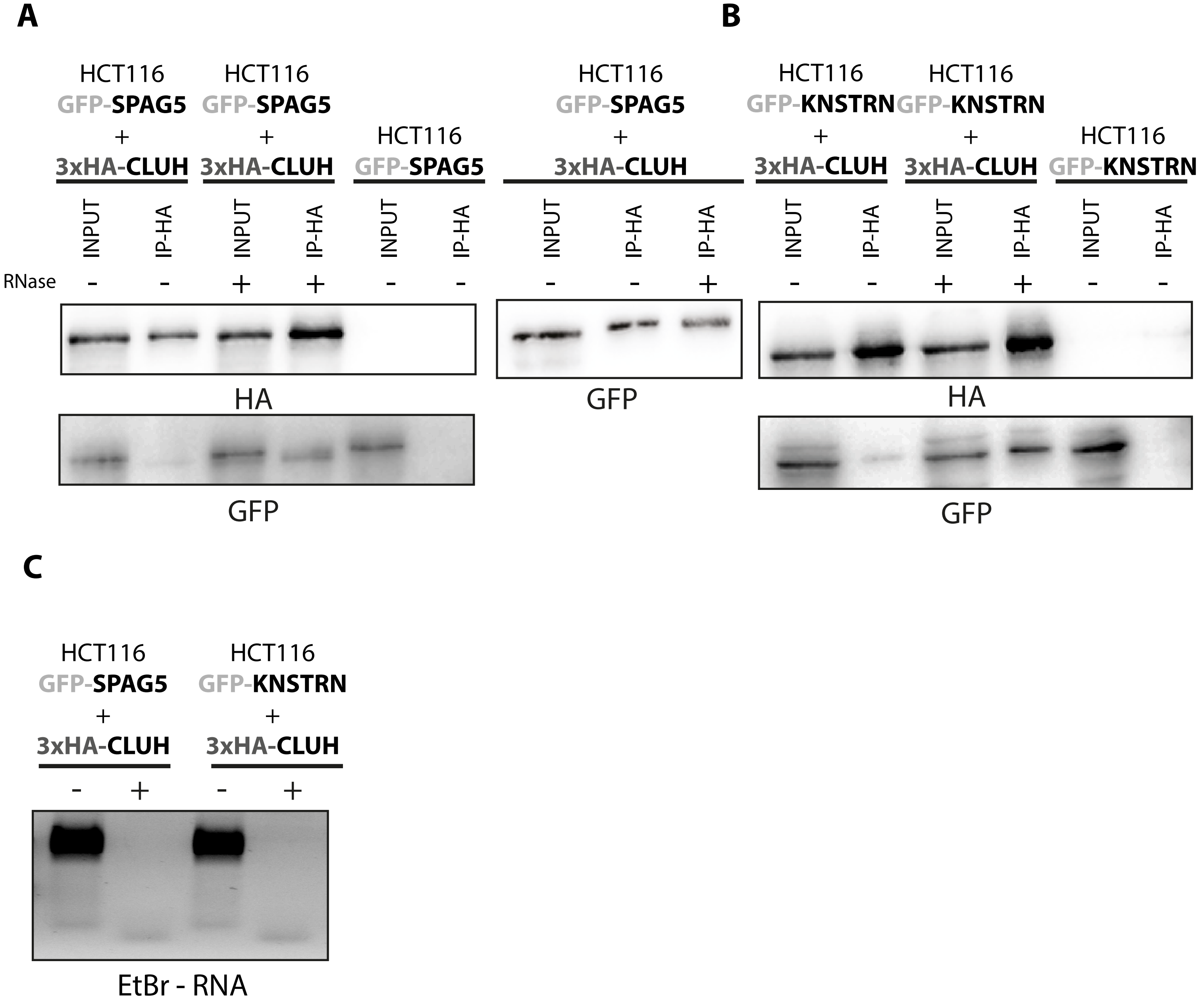

A
B
HCT116
GFP-SPAG5
+
3xHA-CLUH
HCT116
GFP-SPAG5
+
3xHA-CLUH
HCT116
GFP-SPAG5
HCT116
GFP-SPAG5
+
3xHA-CLUH
HCT116
GFP-KNSTRN
+
3xHA-CLUH
HCT116
GFP-KNSTRN
+
3xHA-CLUH
HCT116
GFP-KNSTRN
INPUT
IP-HA
RNase
-
+
HA
GFP
C
HCT116
GFP-SPAG5
+
3xHA-CLUH
HCT116
GFP-KNSTRN
+
3xHA-CLUH
EtBr - RNA

Figure S2: RNA-independent CLUH interaction with SPAG5 and KNSTRN.
(A-B) Western blot analysis of co-IP, between 3xHA-CLUH and GFP-SPAG5 (**A**) or GFP-tagged KNSTRN (**B**) stably expressed in HCT116 cells. The co-IP is performed using magnetic beads coupled with anti-HA antibodies (IP-HA) on total extracts (INPUT) treated (+) or not (-) with RNaseA/T1. Proteins are detected using anti-HA and anti-GFP antibodies. The loaded samples correspond to 0.5% of the input and 20% of the pulled-down samples. **(C)** Ethidium bromide-stained agarose gel loaded with RNase treated (+) or non-treated (-) total protein extracts used for the IP showing the presence of ribosomal RNA.
