# Supplementary material for: The interactome of CLUH reveals its association to SPAG5 and its co-translational proximity to mitochondrial proteins
Source: BMC Biol. 2022 Jan 10;20:13. doi: 10.1186/s12915-021-01213-y (PMC8744257; doi:10.1186/s12915-021-01213-y)
Supplement: Supplementary file 5 — Additional file 5:. Figure S3. CLUH-SPAG5 cytoplasmic structures are not stress granules. [file 12915_2021_1213_MOESM5_ESM.pdf]

Figure S3

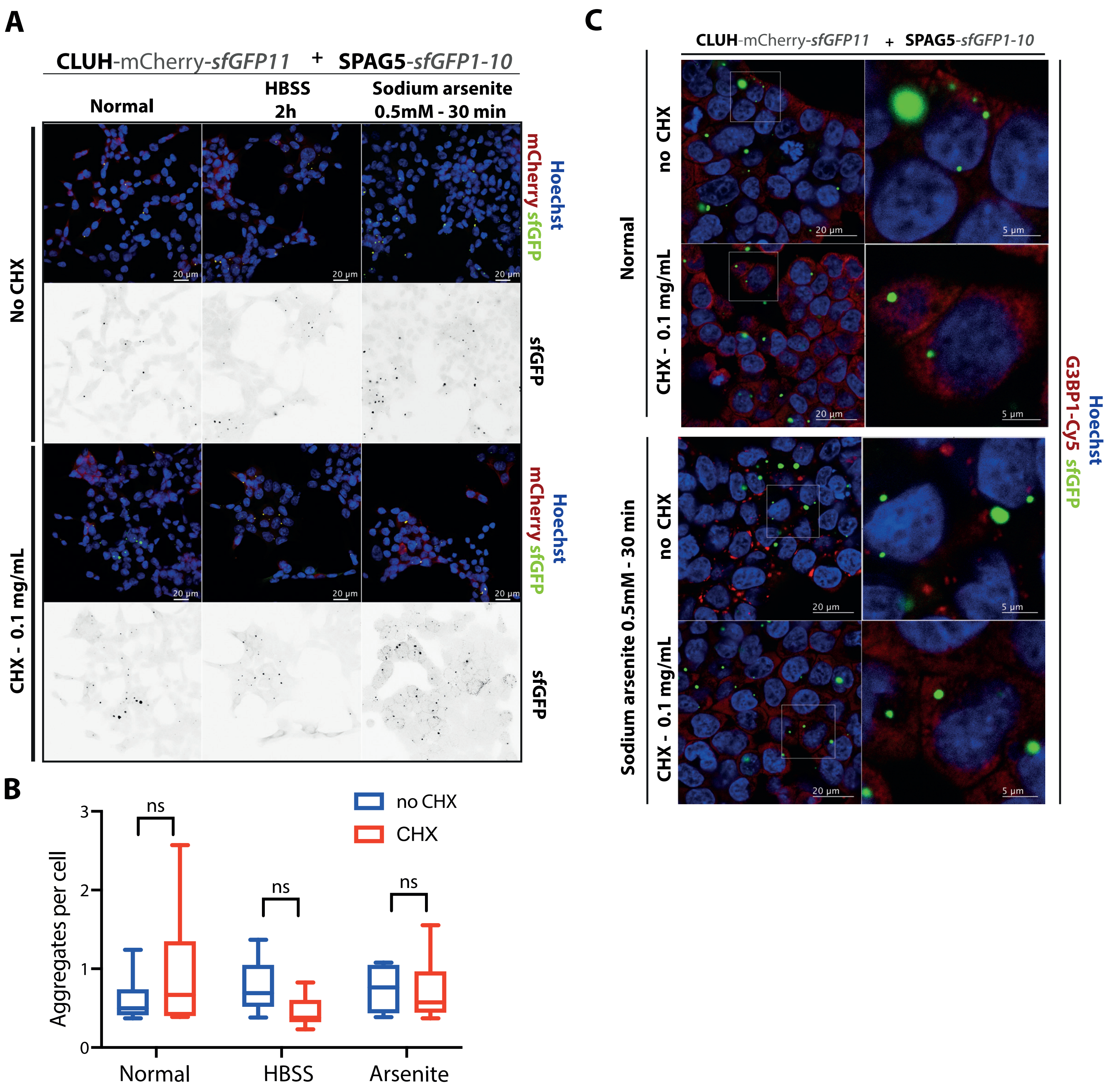
A
CLUH-mCherry-sfGFP11 + SPAG5-sfGFP1-10
Normal
HBSS 2h
Sodium arsenite 0.5mM - 30 min
No CHX
CHX - 0.1 mg/mL
Hoechst mCherry sfGFP
sfGFP
20 µm
B
Aggregates per cell
no CHX
CHX
ns
Normal
HBSS
Arsenite
0
1
2
3
C
CLUH-mCherry-sfGFP11 + SPAG5-sfGFP1-10
Normal
Sodium arsenite 0.5mM - 30 min
no CHX
CHX - 0.1 mg/mL
Hoechst G3BP1-Cy5 sfGFP
20 µm
5 µm

**Figure S3: CLUH-SPAG5 cytoplasmic structures are not stress granules.**
**(A)** Split-GFP analysis of CLUH and SPAG5 interaction on fixed cells expressing both CLUH fused with mCherry-sfGFP11 and SPAG5 fused with sfGFP1-10. The constructs are stably expressed in HCT116 cells cultured in standard conditions (normal), nutritional stress medium for 2 hours (HBSS, low-glucose medium devoid of serum and amino acids) and oxidative stress condition for 30 minutes (0.5 mM sodium arsenite). Treatment with 0.1 mg/mL of cycloheximide (CHX) or control (No CHX) is shown. The reconstituted sfGFP signal is shown in green (upper panels) and in black (lower panels). mCherry signal is shown in red and nuclei, stained with Hoechst, are in blue. **(B)** Quantification of the sfGFP signal aggregates detected with (red) or without (blue) CHX treatment in normal, HBSS and sodium arsenite conditions. Box plots represent the mean aggregate count per cells from 6 different images containing from 40 to 200 cells each. The quantification is performed using the ImageJ AggreCount macro [39]. The statistical non significance (ns) of the difference due to CHX treatment is determined using a t-test with a threshold p-value of 0.05. **(C)** Immunofluorescence analysis of Split-GFP experiment on fixed HCT116 cells stably expressing both CLUH-mCherry-sfGFP11 and SPAG5-sfGFP1-10 constructs. The cells cultured in standard conditions (normal) and oxidative stress conditions (Sodium arsenite) for 30 minutes are treated or not with CHX. The signal of marker G3BP1 is revealed using a specific antibody and secondary antibodies coupled with Cy5 and shown in red. The mCherry signal in not shown. The reconstituted sfGFP signal is shown in green and nuclei, stained with Hoechst, are in blue.
