# Supplementary material for: The interactome of CLUH reveals its association to SPAG5 and its co-translational proximity to mitochondrial proteins
Source: BMC Biol. 2022 Jan 10;20:13. doi: 10.1186/s12915-021-01213-y (PMC8744257; doi:10.1186/s12915-021-01213-y)
Supplement: Supplementary file 6 — Additional file 6:. Figure S4. SPAG5 is not required for CLUH-self interaction. [file 12915_2021_1213_MOESM6_ESM.pdf]

Figure S4

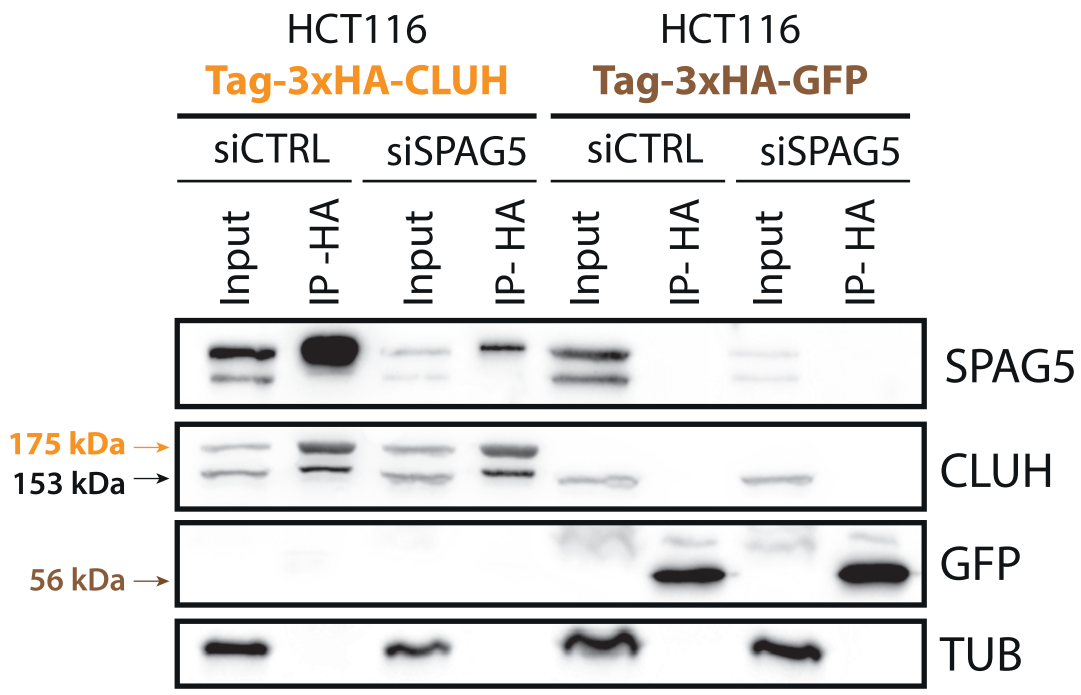
HCT116
Tag-3xHA-CLUH
HCT116
Tag-3xHA-GFP
siCTRL
siSPAG5
siCTRL
siSPAG5
Input
IP -HA
Input
IP- HA
Input
IP- HA
Input
IP- HA
SPAG5
175 kDa
153 kDa
CLUH
56 kDa
GFP
TUB

Figure S4: SPAG5 is not required for CLUH-self interaction.

Western blot analysis of CLUH self-interaction and its interaction with SPAG5 by co-IP. HCT116 cells stably expressing BioID2-3xHA-CLUH (Tag-3xHA-CLUH) protein are transfected with siRNA directed against SPAG5 (siSPAG5) or with non-targeting control siRNA (siCTRL). HCT116 expressing identically tagged GFP protein (Tag-3xHA-GFP) is used as a control. The co-IP is performed on total protein extracts (INPUT) using magnetic beads couplet with anti-HA antibodies (IP-HA). The loaded samples correspond to 0.5% of the input and 20% of the pulled-down samples. The indicated proteins are revealed using specific antibodies. The molecular weight of the tagged CLUH (orange), the endogenous CLUH (black) and GFP (brown) is indicated. TUBULIN (TUB) is used as a loading control.
