# Supplementary material for: The interactome of CLUH reveals its association to SPAG5 and its co-translational proximity to mitochondrial proteins
Source: BMC Biol. 2022 Jan 10;20:13. doi: 10.1186/s12915-021-01213-y (PMC8744257; doi:10.1186/s12915-021-01213-y)
Supplement: Supplementary file 8 — Additional file 8:. Figure S5. Identification of CLUH proximal proteins using BioID in undifferentiated and EB differentiated mESCs. [file 12915_2021_1213_MOESM8_ESM.pdf]

Figure S5

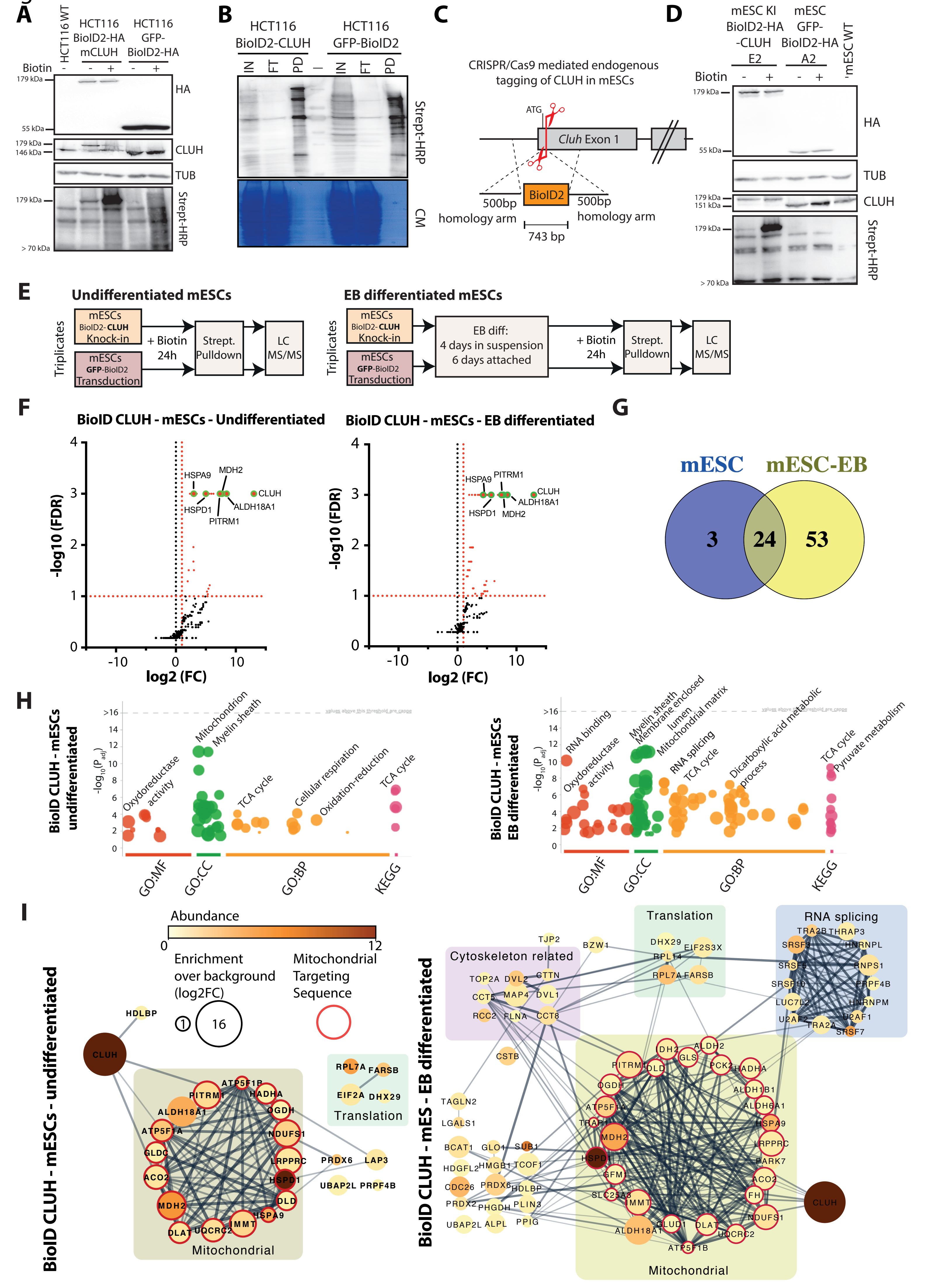

A
HCT116 WT
HCT116 BioID2-HA mCLUH
HCT116 GFP-BioID2-HA
Biotin
HA
CLUH
TUB
Strept-HRP
B
HCT116 BioID2-CLUH
HCT116 GFP-BioID2
IN FT PD
Strept-HRP
CM
C
CRISPR/Cas9 mediated endogenous tagging of CLUH in mESCs
ATG
Cluh Exon 1
BioID2
500bp homology arm
500bp homology arm
743 bp
D
mESC KI BioID2-HA -CLUH E2
mESC GFP-BioID2-HA A2
mESC WT
Biotin
HA
TUB
CLUH
Strept-HRP
E
Undifferentiated mESCs
EB differentiated mESCs
Triplicates
mESCs BioID2-CLUH Knock-in
mESCs GFP-BioID2 Transduction
+ Biotin 24h
Strept. Pulldown
LC MS/MS
EB diff: 4 days in suspension 6 days attached
F
BioID CLUH - mESCs - Undifferentiated
BioID CLUH - mESCs - EB differentiated
-log10 (FDR)
log2 (FC)
HSPA9 MDH2 CLUH HSPD1 ALDH18A1 PITRM1
G
mESC
mESC-EB
3
24
53
H
BioID CLUH - mESCs undifferentiated
BioID CLUH - mESCs EB differentiated
GO:MF GO:CC GO:BP KEGG
Oxydoreductase activity
Mitochondrion
Myelin sheath
TCA cycle
Cellular respiration
Oxidation-reduction
RNA binding
Membrane enclosed lumen
Mitochondrial matrix
RNA splicing
Dicarboxylic acid metabolic process
Pyruvate metabolism
I
Abundance
Enrichment over background (log2FC)
Mitochondrial Targeting Sequence
BioID CLUH - mESCs - undifferentiated
BioID CLUH - mES - EB differentiated
Mitochondrial
Translation
Cytoskeleton related
RNA splicing

Figure S5: Identification of CLUH proximal proteins using BioID in undifferentiated and EB differentiated mESCs.

(A) Western blot analysis of the expression of BioID2-HA-CLUH and GFP-BioID2-HA proteins in HCT116 derived polyclonal stable cell line. Total extract from wild-type HCT116 cells (HCT116 WT) is used a control. CLUH and Tubulin (TUB) are revealed using specific antibodies. The cells were incubated in the presence of 50µM biotin for 24h and global biotinylation activity revealed using HRP-coupled streptavidin (Strept-HRP). **(B)** Representative image of the western blot analysis of one replicate BioID experiment performed on HCT116 cells and send for LC-MS/MS analysis. Equal volumes of both the input protein extract (IN) and the flow through (FT) as well as a 1/10 fraction of the pulldown (PD) sample are analyzed. The biotinylated proteins are revealed using HRP-coupled streptavidin (Strept-HRP). The Coomassie staining of the membrane (CM) is used as loading control. **(C)** Schematic representation of the CRISPR/Cas9 mediated knock-in strategy in mESCs to endogenously tag CLUH in N-terminal with BioID2-HA. Red scissors indicate the cleavage sites **(D)** Western blot analysis of the expression of BioID2-HA-CLUH in CRISPR/Cas9 generated E2 knock-in clone and of the expression of GFP-BioID2 protein in mESCs (E14) derived stable cell line. Total extract from wild-type ESCs (mESCs WT) is used a control. CLUH and Tubulin (TUB) are revealed using specific antibodies. The cells were incubated in the presence of 50µM biotin for 24h and global biotinylation activity revealed using HRP-coupled streptavidin. **(E)** Schematic representation of the BioID experimental design using undifferentiated (left) and embryoïd bodies (EB) differentiated (right) mESCs expressing the BioID2 protein fused to CLUH (endogenous tagging, clone E2) or to GFP (stable cell line generated by lentiviral transduction) proteins. The proximity labeling is performed for 24 hours in the presence of 50 µM biotin in the medium. Biotinylated proteins, from both the specific (BioID2-CLUH) and control (GFP-BioID2) samples, are isolated using streptavidin-coupled magnetic beads and identified by Liquid Chromatography coupled to tandem Mass Spectrometry (LC MS/MS). Full dataset and analysis are available in Table S4 and Table S5. **(F)** Volcano plots showing the global enrichment of proteins in BioID2-CLUH versus the GFP-BioID2 control in both undifferentiated (left) and EB differentiated (right) mESCs. The x-axis shows the log2 fold change (FC), and the y-axis shows the −log10 of the false discovery rate (n=3), obtained using SAINTexpress software [26]. Significantly enriched proteins are shown in red and are defined by a fold change greater than two and a FDR < 0.1 (shown as dashed red lines). CLUH and five of the most abundant mitochondrial proteins are labeled and identified with a green circle. **(G)** Venn diagram showing the intersection of CLUH proximal proteins, identified by BioID, in undifferentiated (mESCs) and EB differentiated cells (EB). **(H)** Manhattan plot illustrating the gene ontology and pathway enrichment analysis of proteins identified in BioID experiment on undifferentiated (left) and differentiated (right) mESCs, generated using g:profiler tool [43]. The functional terms, associated with the protein lists, are grouped in four categories: GO: MF (Molecular Function), GO: CC (Cellular Component), GO: BP (Biological Process) and KEGG pathways. The y-axis shows the adjusted enrichment p-values in negative log10 scale. The circle sizes are in accordance with the corresponding term size (in the database) and terms from the same GO subtree are located close to each other on the x-axis. The more significantly enriched terms are labeled. **(I)** Visualization of the functional interaction network of CLUH proximal proteins identified by BioID on undifferentiated (left) and EB differentiated (right) mESCs, generated using the Cytoscape StringApp [44]. The proteins have been grouped according to the most represented functional categories: “Cytoskeleton related”, “Translation”, “Mitochondrial” and “RNA splicing”. The confidence score of each interaction is mapped to the edge thickness

and opacity. The size of the node relates to the enrichment in log2 fold change (log2FC) over the BioID-GFP background control. The protein abundance in the BioID2-CLUH sample is illustrated by the color scale and corresponds to the specific spectral count normalized to the protein size. Proteins with mitochondrial targeting sequences (MTS) according to Uniprot database are highlighted in red.
