# Supplementary material for: The interactome of CLUH reveals its association to SPAG5 and its co-translational proximity to mitochondrial proteins
Source: BMC Biol. 2022 Jan 10;20:13. doi: 10.1186/s12915-021-01213-y (PMC8744257; doi:10.1186/s12915-021-01213-y)
Supplement: Supplementary file 12 — Additional file 12: Figure S6. Generation of CRISPR/Cas9 knockout cells for CLUH and BioID protein coverage. [file 12915_2021_1213_MOESM12_ESM.pdf]

# Figure S6

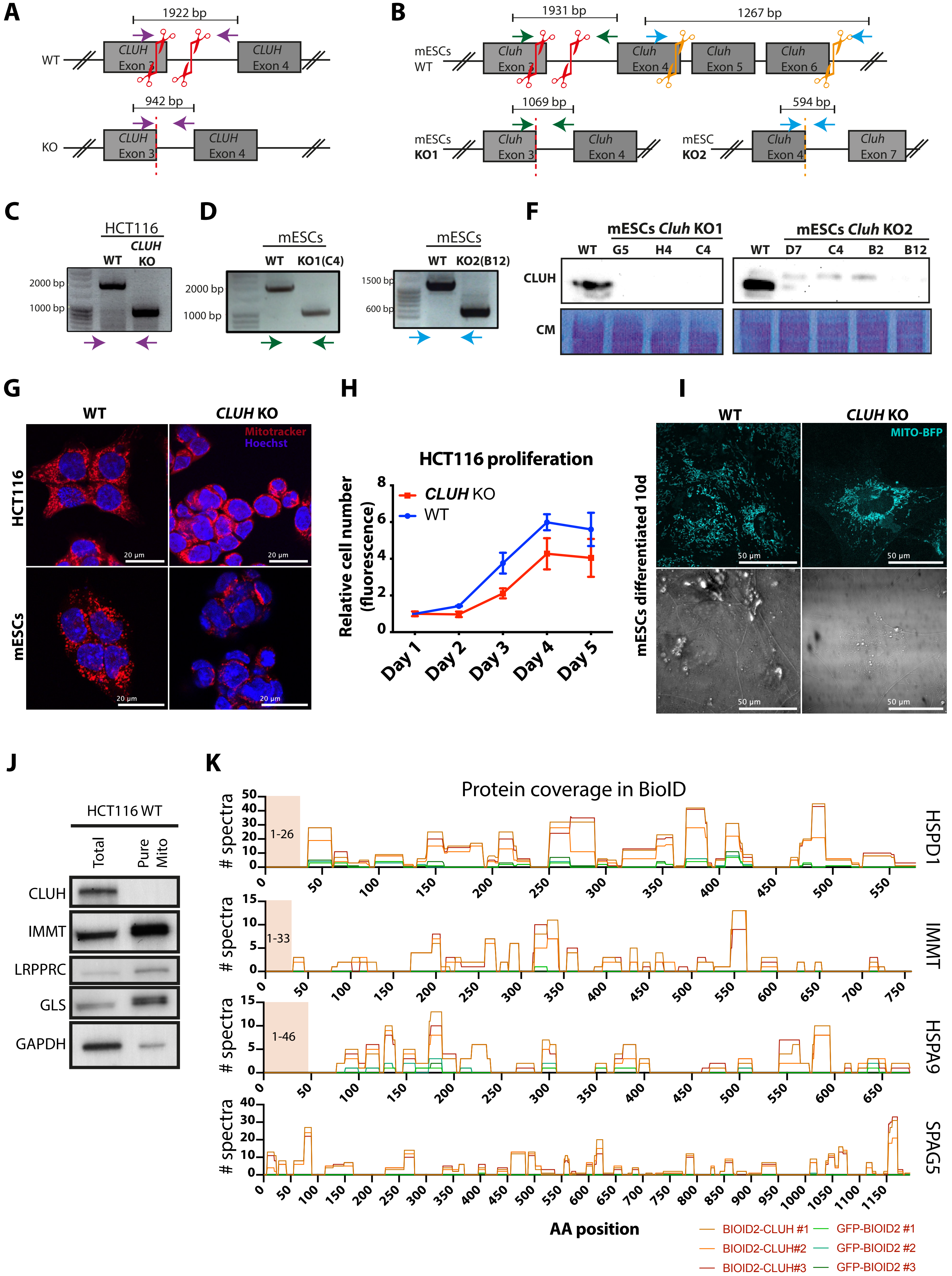
A
1922 bp
WT
CLUH Exon 3
CLUH Exon 4
942 bp
KO
CLUH Exon 3
CLUH Exon 4
B
1931 bp
1267 bp
mESCs WT
Cluh Exon 3
Cluh Exon 4
Cluh Exon 5
Cluh Exon 6
1069 bp
mESCs KO1
Cluh Exon 3
Cluh Exon 4
594 bp
mESC KO2
Cluh Exon 4
Cluh Exon 7
C
HCT116
WT
CLUH KO
2000 bp
1000 bp
D
mESCs
WT
KO1(C4)
2000 bp
1000 bp
mESCs
WT
KO2(B12)
1500 bp
600 bp
F
mESCs Cluh KO1
WT G5 H4 C4
mESCs Cluh KO2
WT D7 C4 B2 B12
CLUH
CM
G
WT
CLUH KO
Mitotracker
Hoechst
HCT116
mESCs
20 µm
H
HCT116 proliferation
CLUH KO
WT
Relative cell number (fluorescence)
8
6
4
2
0
Day 1
Day 2
Day 3
Day 4
Day 5
I
WT
CLUH KO
MITO-BFP
mESCs differentiated 10d
50 µm
J
HCT116 WT
Total
Pure Mito
CLUH
IMMT
LRPPRC
GLS
GAPDH
K
Protein coverage in BioID
# spectra
1-26
HSPD1
1-33
IMMT
1-46
HSPA9
SPAG5
AA position
BIOID2-CLUH #1
BIOID2-CLUH#2
BIOID2-CLUH#3
GFP-BIOID2 #1
GFP-BIOID2 #2
GFP-BIOID2 #3

**Figure S6: Generation of CRISPR/Cas9 knockout cells for *CLUH* and BioID protein coverage.** **(A-B)** Schematic representation of the CRISPR/Cas9 mediated knock-out of *CLUH* in HCT116 **(A)** and mESCs **(B).** A paired sgRNA strategy is used to delete DNA fragments leading to gene inactivation*.* Red scissors indicate the cleavage site of each sgRNA and colored arrows show the location of PCR genotyping primers. PCR amplification products size are indicated for both wild-type cells and each mutant. **(C-D)** Agarose gel showing the genotyping PCR results of WT and mutant HCT116 **(C)** and mESCs **(D)**. The used PCR primers match the color code indicated in **(A, B). (F)** Western blot showing the expression of *Cluh* in selected mESC *Cluh* KO clones. CLUH is detected using specific antibodies. Coomassie staining of the membrane (CM) is used as loading control. **(G)** Confocal microscopy images of mESC and HCT116 *CLUH* KO cells. Parental wildtype cells are also shown. The mESC *Cluh* KO1 (C4) clone is shown. Mitochondria (red) are labeled using MitoTracker™ Red CMXRos. Nuclei (blue) are stained with Hoechst. The scale bar is indicated in white. **(H)** Proliferation assay performed on HCT116 *CLUH* KO cells and WT cells over 5 days. The relative cell number is measured compared to day1, using a florescence assay (CellTiter-FluorTM, Promega). The error bars correspond to the standard deviation of three biological replicate experiments. **(I)** Confocal microscopy images of both WT and *Cluh* KO (C4) mESCs stably expressing mitochondrial BFP protein (fusion with Cox8a mitochondrial targeting sequence). Mitochondria are shown in light blue (upper panels) and transmitted light images are shown in gray. The scale bar is indicated in white. **(J)** Western blot on total and "pure" mitochondrial fractions from wild-type HCT116 cells. Indicated proteins are revealed using specific antibodies. **(K)** Plot showing the protein coverage from LC MS/MS identification of the most abundant proteins from CLUH BioID experiment on HCT116 cells (see Figure 4). The x-axis corresponds to the amino acid (aa) position for each protein and the y-axis show specific spectral counts for both BioID2-CLUH and GFP-BIOID2 samples. All replicate samples are indicated with a color scale. The N-terminal region of the proteins, containing the mitochondrial targeting peptide, according to Uniprot annotations, is highlighted in orange shadow.
