# Supplementary material for: The interactome of CLUH reveals its association to SPAG5 and its co-translational proximity to mitochondrial proteins
Source: BMC Biol. 2022 Jan 10;20:13. doi: 10.1186/s12915-021-01213-y (PMC8744257; doi:10.1186/s12915-021-01213-y)
Supplement: Supplementary file 13 — Additional file 13:. Figure S7. Identification of CLUH proximal proteins in HCT116 cells using a TurboID time course approach. [file 12915_2021_1213_MOESM13_ESM.pdf]

Figure S7

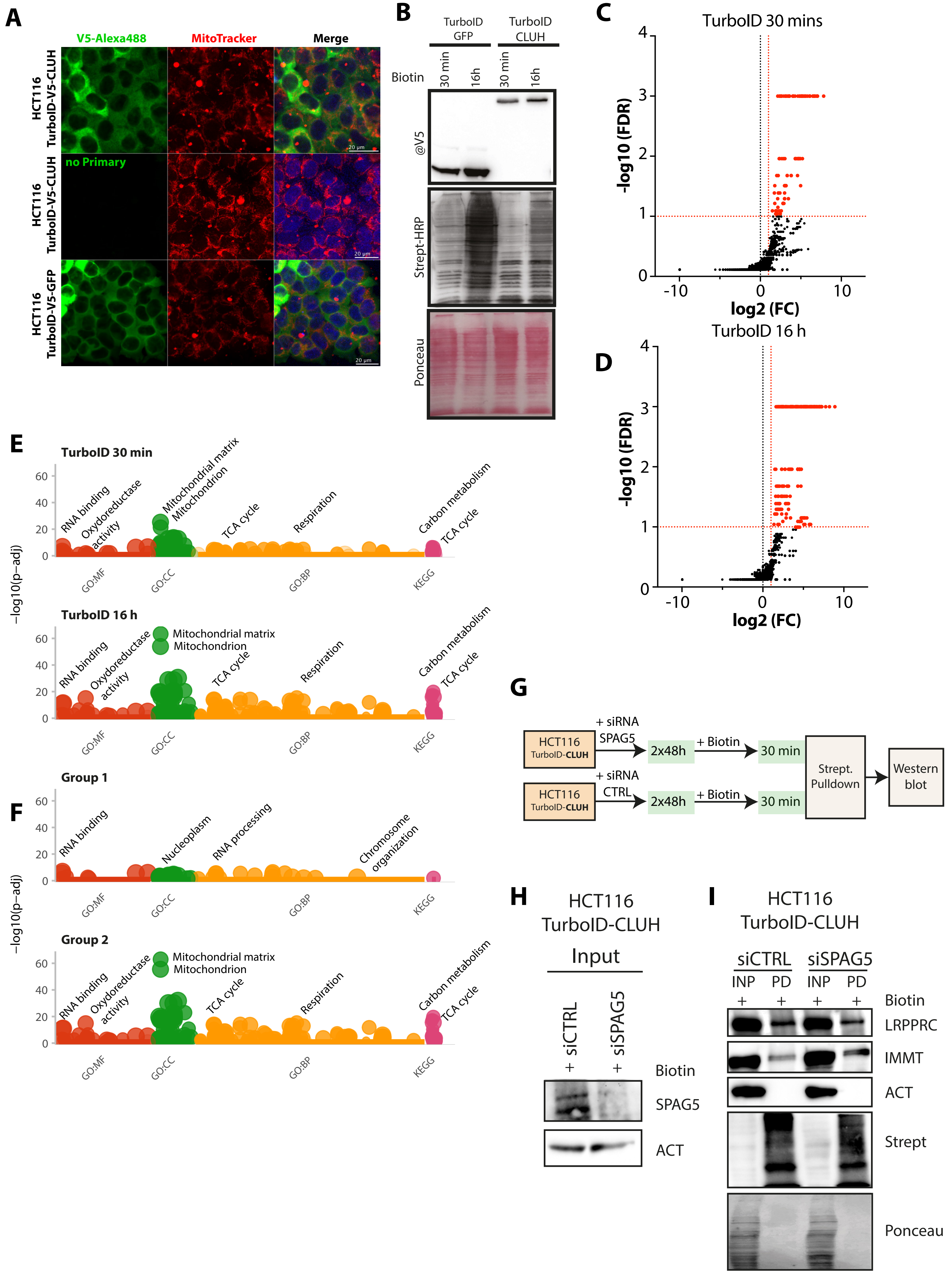
A
V5-Alexa488
MitoTracker
Merge
HCT116 TurboID-V5-CLUH
no Primary
HCT116 TurboID-V5-CLUH
HCT116 TurboID-V5-GFP
20 µm
B
TurboID GFP
TurboID CLUH
Biotin
30 min
16h
30 min
16h
@V5
Strept-HRP
Ponceau
C
TurboID 30 mins
-log10 (FDR)
log2 (FC)
D
TurboID 16 h
-log10 (FDR)
log2 (FC)
E
TurboID 30 min
TurboID 16 h
−log10(p−adj)
RNA binding
Oxydoreductase activity
Mitochondrial matrix
Mitochondrion
TCA cycle
Respiration
Carbon metabolism
GO:MF
GO:CC
GO:BP
KEGG
F
Group 1
Group 2
Nucleoplasm
RNA processing
Chromosome organization
G
HCT116 TurboID-CLUH
+ siRNA SPAG5
+ siRNA CTRL
2x48h
+ Biotin
30 min
Strept. Pulldown
Western blot
H
HCT116 TurboID-CLUH
Input
siCTRL
siSPAG5
Biotin
SPAG5
ACT
I
HCT116 TurboID-CLUH
siCTRL
siSPAG5
INP
PD
Biotin
LRPPRC
IMMT
ACT
Strept
Ponceau

Figure S7: Identification of CLUH proximal proteins in HCT116 cells using a TurboID time course approach.

(A) Confocal microscopy images of HCT116 cells stably expressing TurboID-V5-CLUH or TurboID-V5-GFP fusion proteins. The proteins are detected using anti-V5 primary antibody and revealed using Alexa488 secondary antibody (green). Mitochondria (red) are labeled using MitoTracker™ Red CMXRos. Nuclei (blue) are stained with Hoechst. The scale bar is indicated in white. **(B)** Western blot showing the expression of TurboID-GFP and TurboID-CLUH constructs stably expressed in HCT116 cells, at 30 min and 16h after the addition of biotin in the medium (Figure 5A). The proteins are revealed using anti-V5 antibodies. Biotinylated proteins are revealed using HRP-coupled streptavidin. Ponceau staining of the membrane is shown as loading control. **(C-D)** Volcano plots showing the global enrichment of proteins in TurboID-CLUH versus the TurboID-GFP control at 30 minutes **(C)** and 16 hours **(D)**. The x-axis shows the log2 fold change (FC), and the y-axis shows the −log10 of the false discovery rate (n=3), obtained using SAINTexpress software [26]. Significantly enriched proteins are shown in red and are defined by a fold change greater than two and a FDR < 0.1 (shown as dashed red lines). **(E-F)** Manhattan plots illustrating the gene ontology and pathway enrichment analysis of proteins identified in TurboID experiment. The analysis is done on all significantly enriched proteins identified at 30 minutes and 16 hours **(E)** as well as for the proteins from group 1 and group 2 **(F)** **(**from figure 5B). The plot is generated using g:profiler tool [43]. The functional terms, associated with the protein lists, are grouped in four categories: GO: MF (Molecular Function), GO: CC (Cellular Component), GO: BP (Biological Process) and KEGG pathways. The y-axis shows the adjusted enrichment p-values in negative log10 scale. The circle sizes are in accordance with the term size (i.e. larger terms have larger circles) and terms from the same GO subtree are located close to each other on the x-axis. The more significantly enriched terms are labeled. **(G)** Schematic representation of the TurboID experimental design using HCT116 cells stably expressing the TurboID protein fused to CLUH in SPAG5 knockdown conditions. Cells are transfected with non-specific (siCTRL) or SPAG5 specific (siSPAG5) siRNAs, two times at 48 hours intervals prior to biotin pulse labeling. The proximity labeling is performed for 30 minutes in the presence of 50 µM biotin in the culture medium. **(H)** Western blot showing the expression level of SPAG5 in the input samples. **(I)** Streptavidin enriched proteins are analyzed by western blot. Biotinylated proteins are pulled down (PD) from total input extracts (INP) using streptavidin-coupled magnetic beads. The loaded samples correspond to 0.5% of the input and 20% of the pulled-down samples. The CPMPs (LRPPRC, IMMT) and other indicated proteins are revealed using specific antibodies. Biotinylated proteins are revealed using HRP-coupled streptavidin.
