# Supplementary material for: The interactome of CLUH reveals its association to SPAG5 and its co-translational proximity to mitochondrial proteins
Source: BMC Biol. 2022 Jan 10;20:13. doi: 10.1186/s12915-021-01213-y (PMC8744257; doi:10.1186/s12915-021-01213-y)
Supplement: Supplementary file 15 — Additional file 15:. Figure S8. CLUH effect on RNA stability and translation. [file 12915_2021_1213_MOESM15_ESM.pdf]

Figure S8

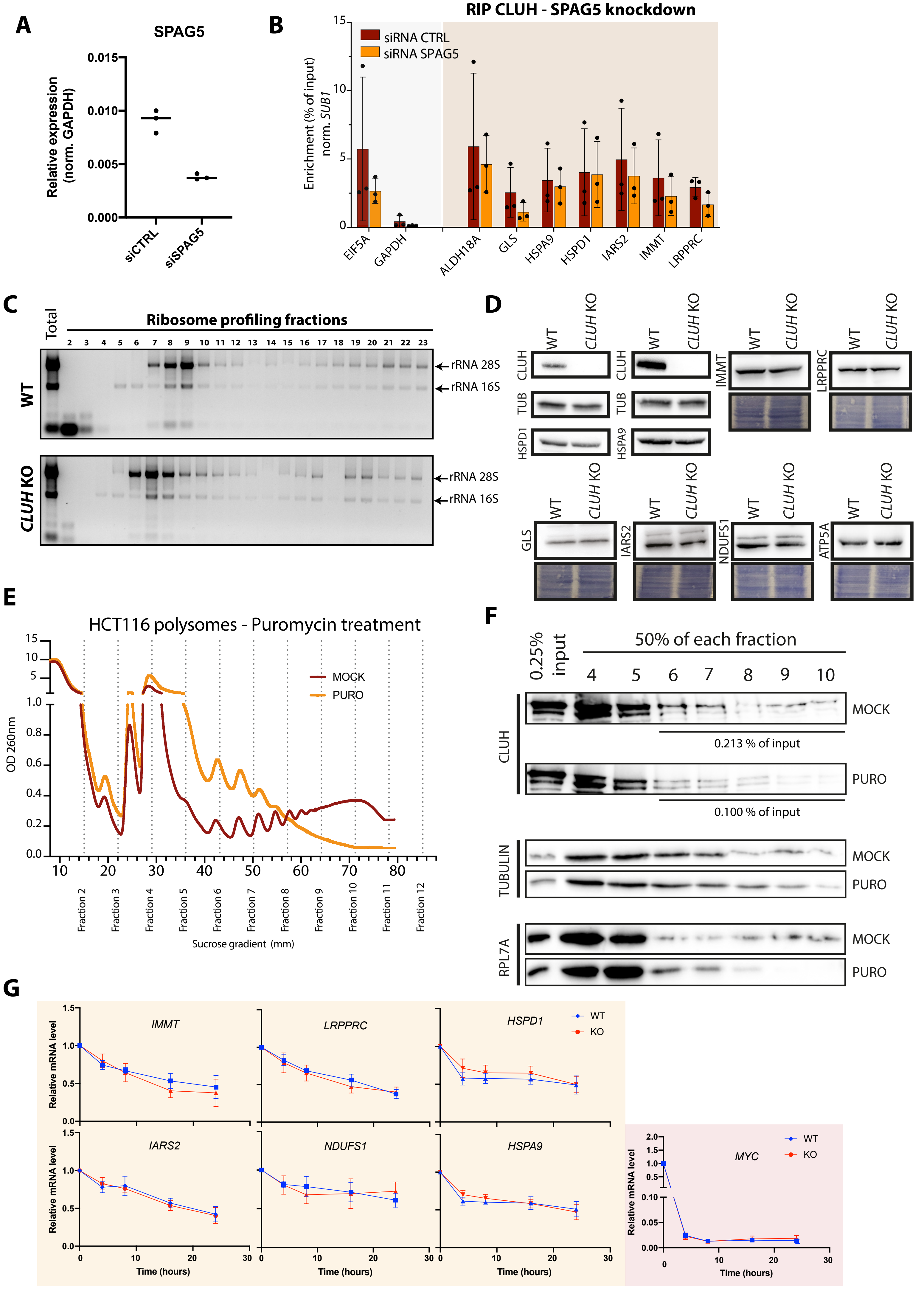

A
SPAG5
Relative expression (norm. GAPDH)
0.015
0.010
0.005
0.000
siCTRL
siSPAG5
B
RIP CLUH - SPAG5 knockdown
siRNA CTRL
siRNA SPAG5
Enrichment (% of input) norm. SUB1
EIF5A
GAPDH
ALDH18A
GLS
HSPA9
HSPD1
IARS2
IMMT
LRPPRC
C
Ribosome profiling fractions
Total
WT
CLUH KO
rRNA 28S
rRNA 16S
D
WT
CLUH KO
CLUH
TUB
HSPD1
HSPA9
IMMT
LRPPRC
GLS
IARS2
NDUFS1
ATP5A
E
HCT116 polysomes - Puromycin treatment
MOCK
PURO
OD 260nm
Sucrose gradient (mm)
Fraction 2
Fraction 3
Fraction 4
Fraction 5
Fraction 6
Fraction 7
Fraction 8
Fraction 9
Fraction 10
Fraction 11
Fraction 12
F
0.25% input
50% of each fraction
MOCK
PURO
0.213 % of input
0.100 % of input
CLUH
TUBULIN
RPL7A
G
IMMT
LRPPRC
HSPD1
IARS2
NDUFS1
HSPA9
MYC
WT
KO
Relative mRNA level
Time (hours)

Figure S8: CLUH effect on RNA stability and translation.

(A) RT-qPCR analysis of SPAG5 expression level in HCT116 cells transfected with transfected with either siRNA directed against SPAG5 (siRNA SPAG5) or non-specific siRNA (siRNA CTRL). The level of SPAG5 normalized GAPDH levels in the three independent knock-down experiment, used for the RIP, are shown. Each dot represents the mean value of each experiment. **(B)** RT-qPCR analysis of RIP experiment performed on wild-type HCT116 cells. The CLUH associated mRNAs are enriched using CLUH-specific antibodies and measured by RT-qPCR. The enrichment of specific mRNA (normalized to *SUB1* levels) is calculated relative to the input sample (% of input). mRNAs coding for CPMPs are highlighted by the orange shadow. The error bars correspond to the standard deviation of three independent experiments. The average value for each replicate is indicated by a dot. **(C)** Representative agarose gel electrophoresis analysis of the RNA extracted from the ribosome profiling fractions of both *CLUH* KO and HCT116 cells. The fractions corresponding to about 3.3 mm of the sucrose gradient are numbered from 1 to 23. Input RNA from total extract (Total) is loaded as control. 28S and 16S ribosomal RNA are indicated. RNA is revealed using ethidium bromide staining. **(D)** Western blot analysis of mitochondrial proteins abundance in total protein extracts from *CLUH* KO and WT HCT116 cells. Indicated proteins are revealed using specific antibodies. TUBULIN (TUB) or Coomassie staining of the membrane is used as loading control. **(E)** Representative graphs of polysome profilings of WT HCT116 cells treated (PURO) or not (MOCK) with 100µg/mL of puromycin. The y-axis corresponds to the absorbance at 260 nm and the x-axis to the distance in the sucrose gradient. The collected polysomal fractions are indicated under the x-axis. **(F)** Representative western blot analysis of polysome profiling of WT HCT116 cells treated (PURO) or not (MOCK) with puromycin. About 50% of fractions 4 to 10 are loaded on the gel. Total input protein extracts corresponding to 0.25% of the input extract used to isolate polysomes are used as controls. Indicated proteins are revealed using specific antibodies. The signal of the heaviest polysomal fractions 6 to 10 is indicated relative to the input levels. **(G)** Analysis of mRNA stability over time, after actinomycin D treatment in *CLUH* KO and WT HCT116 cells. The represented mRNA levels are normalized to *GAPDH* levels and are represented relative to time 0. The error bars represent the standard deviation of three replicate experiments. *MYC* is used as a control for the Actinomycin treatment.
