# Supplementary material for: The interactome of CLUH reveals its association to SPAG5 and its co-translational proximity to mitochondrial proteins
Source: BMC Biol. 2022 Jan 10;20:13. doi: 10.1186/s12915-021-01213-y (PMC8744257; doi:10.1186/s12915-021-01213-y)
Supplement: Supplementary file 16 — Additional file 16:. Figure S9. Polysome profiling from crude mitochondrial extract. [file 12915_2021_1213_MOESM16_ESM.pdf]

Figure S9

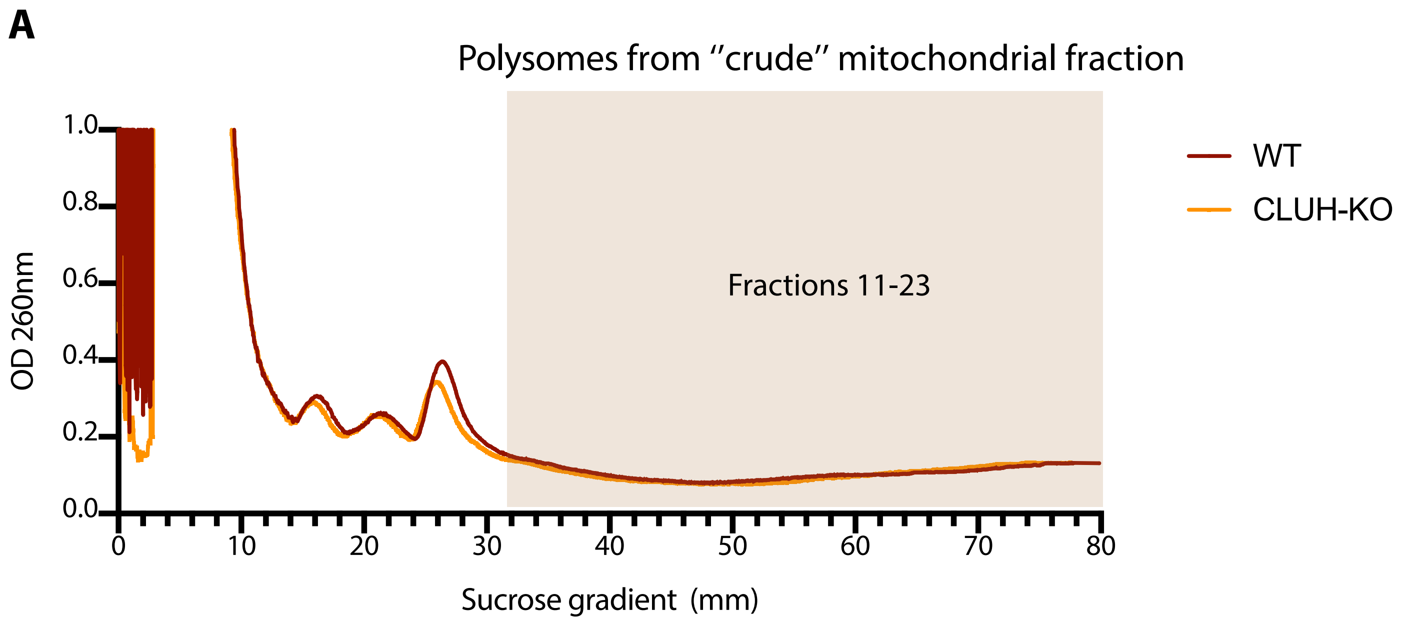
A
Polysomes from "crude" mitochondrial fraction
OD 260nm
1.0
0.8
0.6
0.4
0.2
0.0
0
10
20
30
40
50
60
70
80
Sucrose gradient (mm)
Fractions 11-23
WT
CLUH-KO

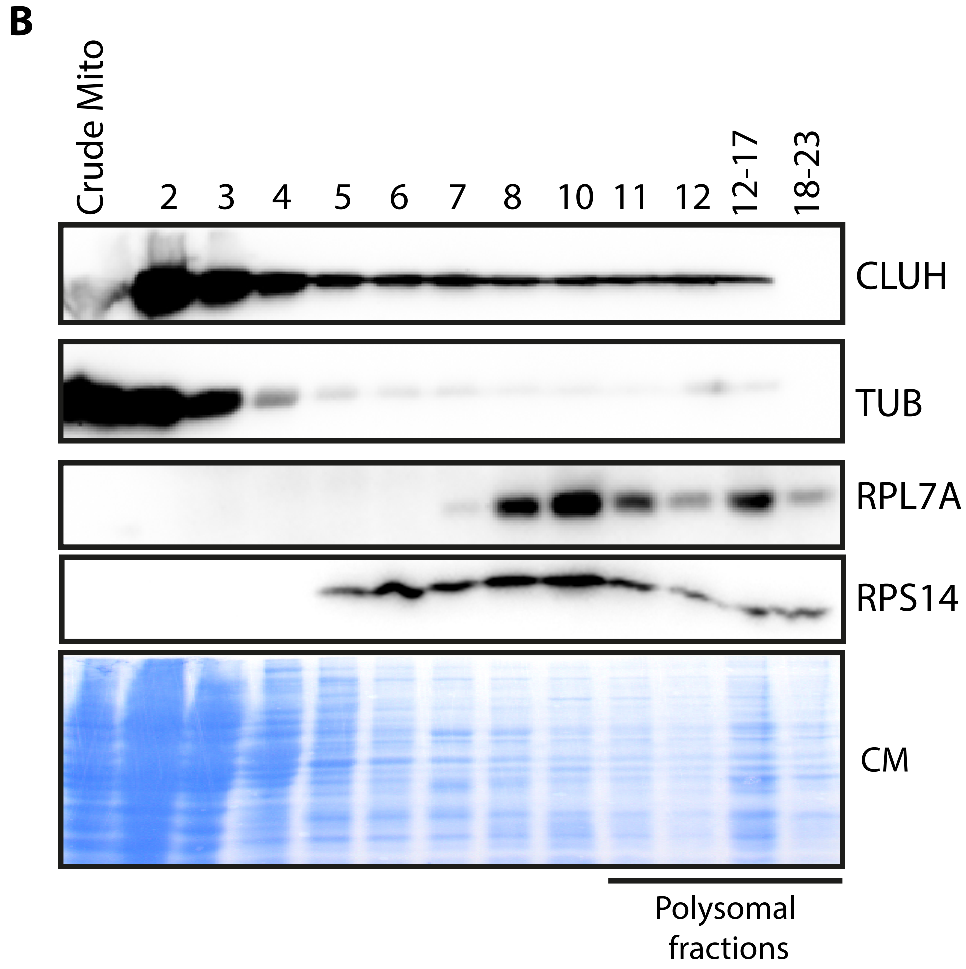
B
Crude Mito
2
3
4
5
6
7
8
10
11
12
12-17
18-23
CLUH
TUB
RPL7A
RPS14
CM
Polysomal fractions

Figure S9: Polysome profiling from crude mitochondrial extract.

(A) Representative graph of polysome profilings from "crude" mitochondrial fractions (see Figure 7D) of WT and *CLUH* KO HCT116 cells. The y-axis corresponds to the absorbance at 260 nm and the x-axis to the distance in the sucrose gradient. The polysomal fractions used for further experiments (Figure 8D) are highlighted in orange shadow. **(B)** Representative western blot analysis of polysome profiling from the crude mitochondrial fraction in WT HCT116 cells. Each fraction corresponds to 3.3 mm of the sucrose gradient and is numbered from 1 to 23. Fractions 12-17 and 18-23 are pooled. Crude mitochondrial extract is used as control. Indicated proteins are revealed using specific antibodies. Coomassie staining of the membrane is shown as loading control. The polysomal fraction used for RNA extraction are underlined.
