# Supplementary material for: The interactome of CLUH reveals its association to SPAG5 and its co-translational proximity to mitochondrial proteins
Source: BMC Biol. 2022 Jan 10;20:13. doi: 10.1186/s12915-021-01213-y (PMC8744257; doi:10.1186/s12915-021-01213-y)
Supplement: Supplementary file 20 — Additional file 20:. supplementary file 1. Uncropped images of western blots. [file 12915_2021_1213_MOESM20_ESM.pdf]

FIGURE 2A

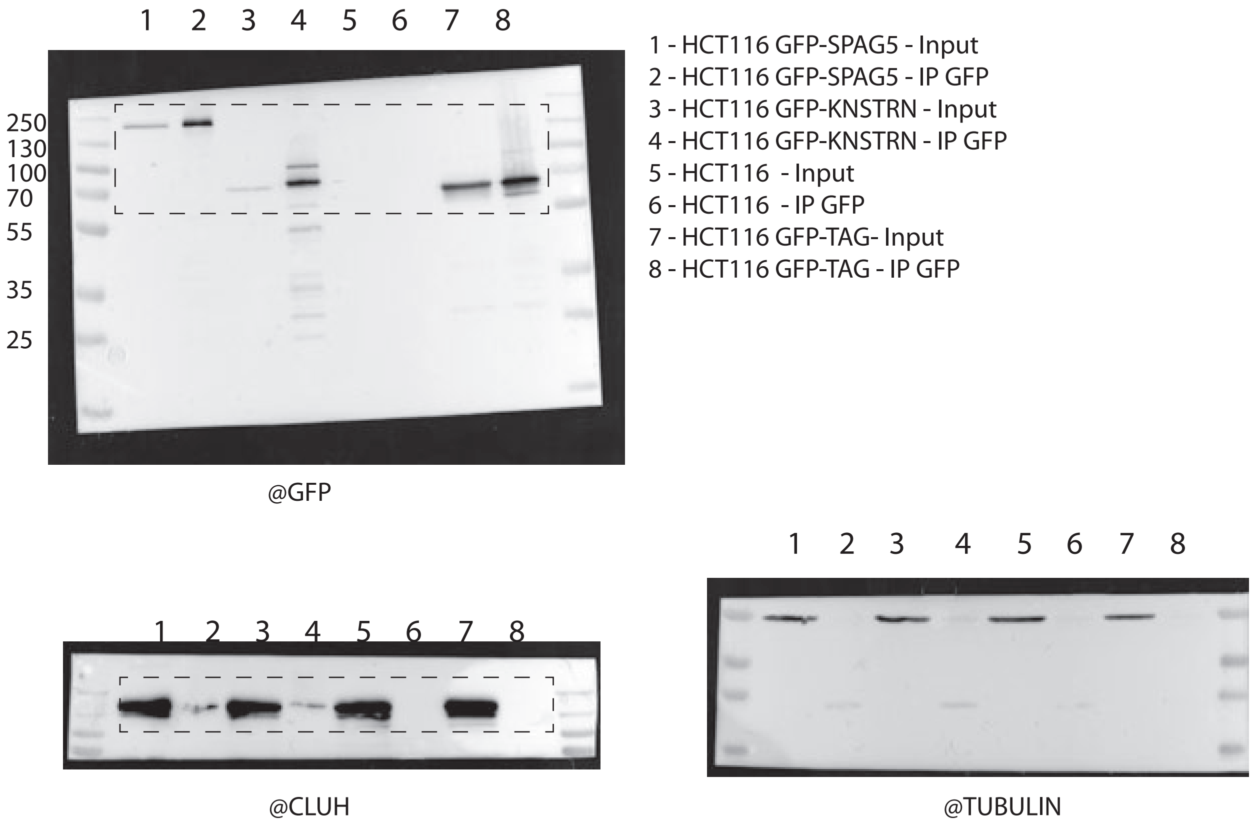
1 2 3 4 5 6 7 8
250
130
100
70
55
35
25
1 - HCT116 GFP-SPAG5 - Input
2 - HCT116 GFP-SPAG5 - IP GFP
3 - HCT116 GFP-KNSTRN - Input
4 - HCT116 GFP-KNSTRN - IP GFP
5 - HCT116 - Input
6 - HCT116 - IP GFP
7 - HCT116 GFP-TAG- Input
8 - HCT116 GFP-TAG - IP GFP
@GFP
1 2 3 4 5 6 7 8
@CLUH
1 2 3 4 5 6 7 8
@TUBULIN

FIGURE 3A

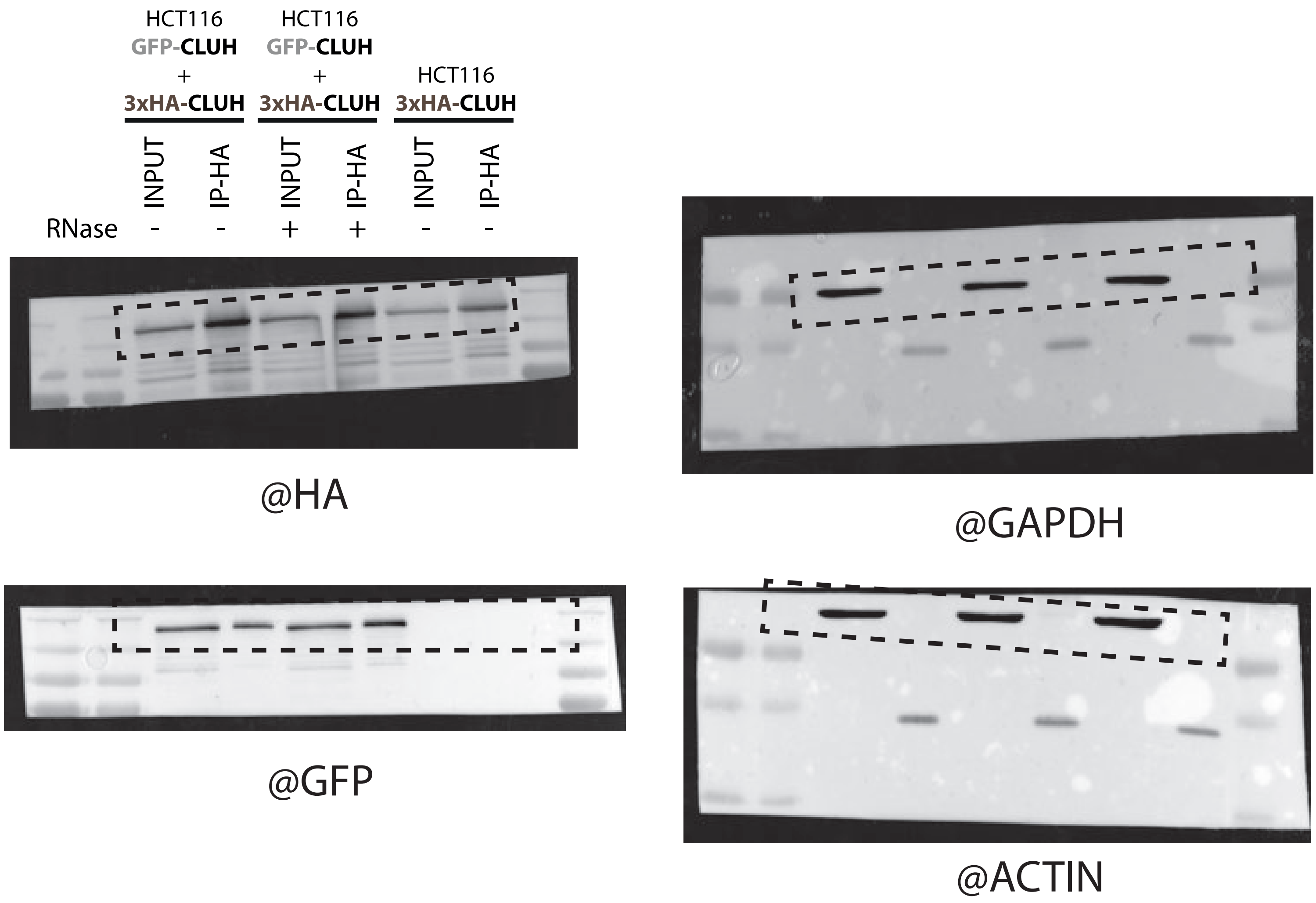

FIGURE 3B

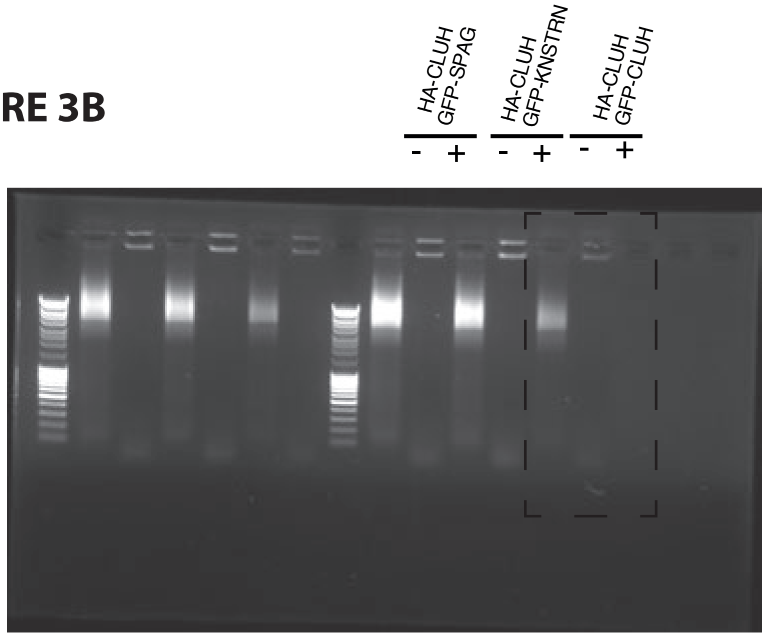

RNAse A/T1 treatment 10 min at 37°C
(20µg/mL RNAse A and 50U/mL of RNAse T1)

AGAROSE GEL RNAse

FIGURE 3D

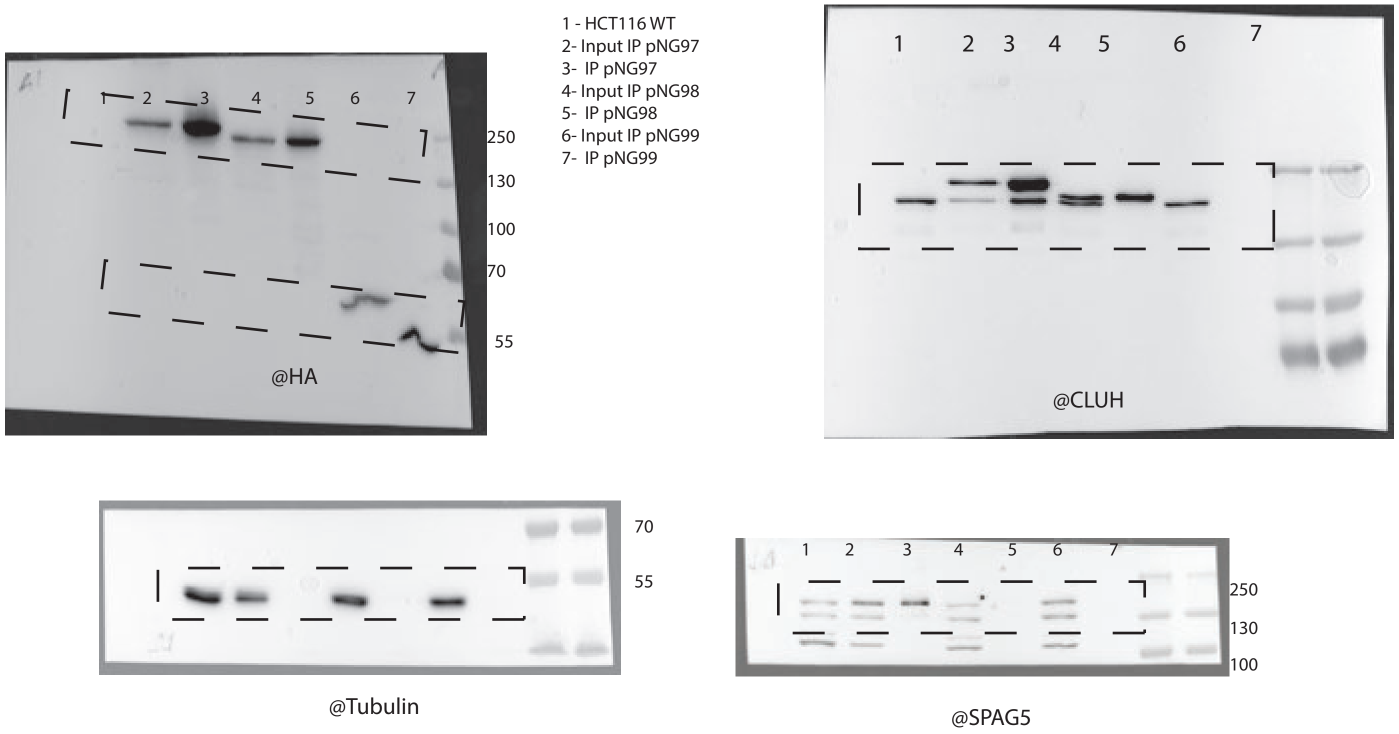
1 - HCT116 WT
2- Input IP pNG97
3- IP pNG97
4- Input IP pNG98
5- IP pNG98
6- Input IP pNG99
7- IP pNG99
1 2 3 4 5 6 7
250
130
100
70
55
@HA
1 2 3 4 5 6 7
@CLUH
70
55
@Tubulin
1 2 3 4 5 6 7
250
130
100
@SPAG5

FIGURE 5A

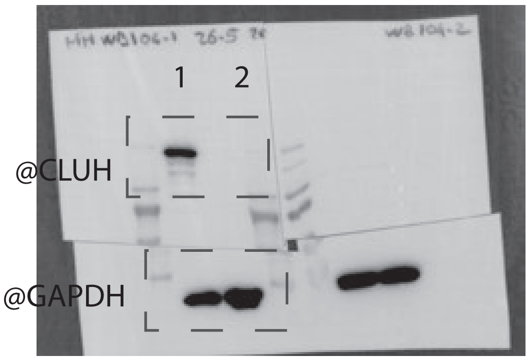

1 - WT HCT116
2 - CLUH KO HCT116

FIGURE 5A

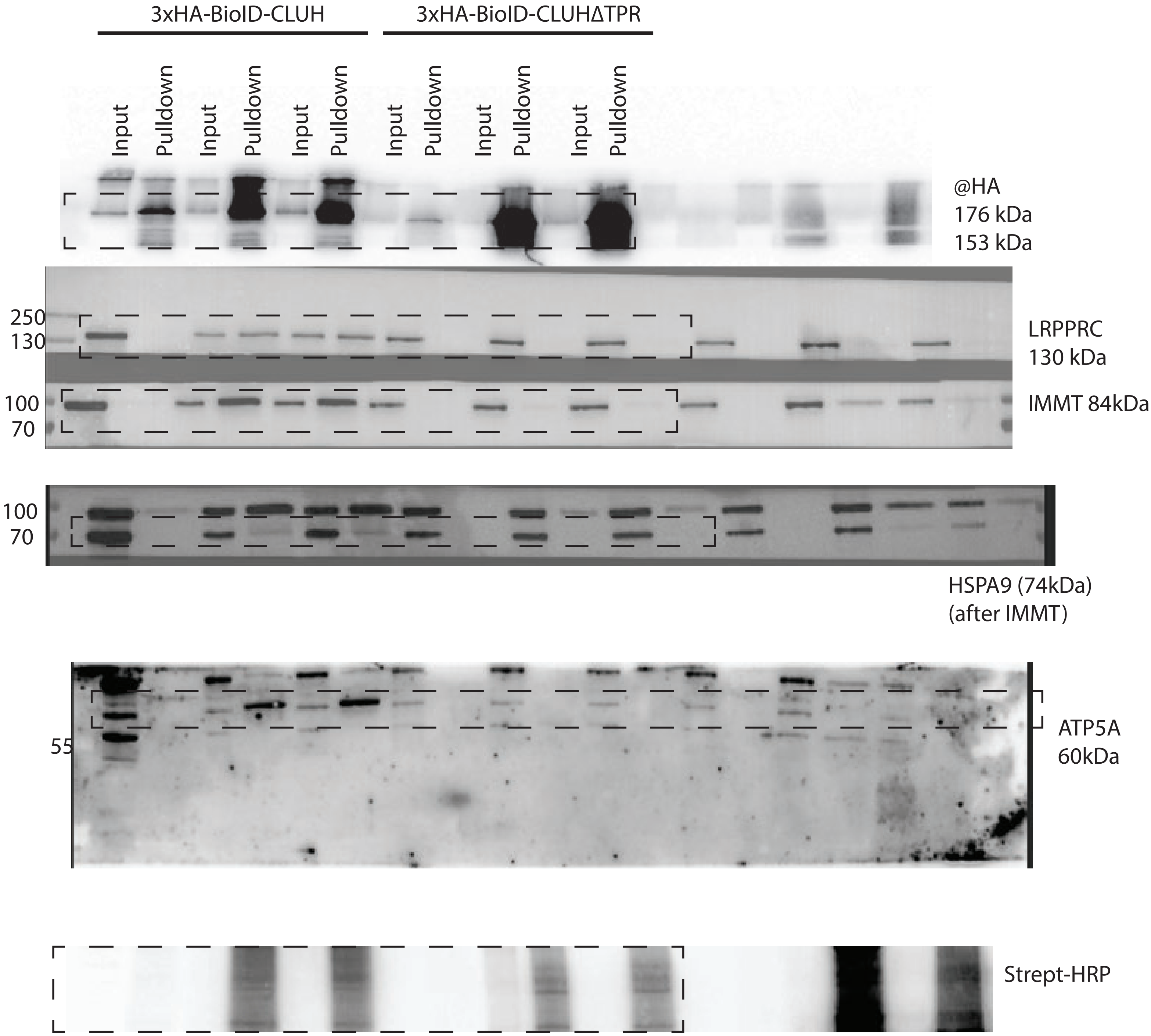

FIGURE 5C

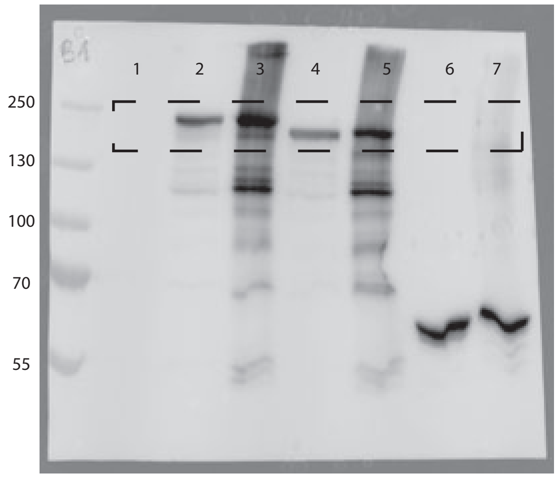

@HA

1 - HCT116 WT
2- Input BioID pNG97
3- Pulldown BioID pNG97
4- Input BioID pNG98
5- IPulldown BioID pNG98
6- Input BioID pNG99
7- Pulldown BioID pNG99

3xHA-BirA-GFP

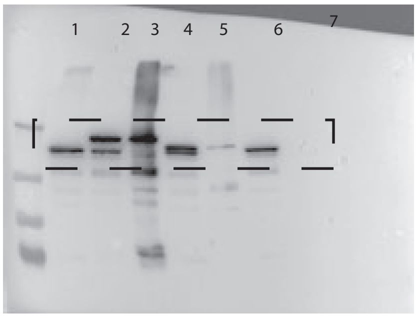

@CLUH

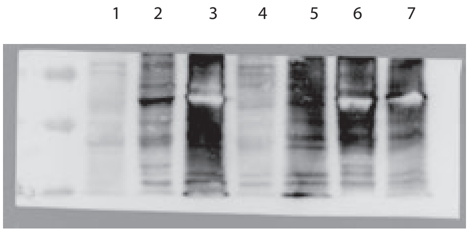

Strepta-HRP

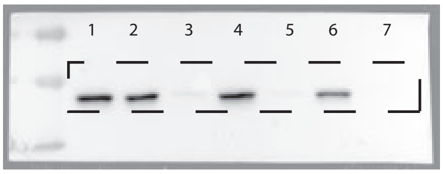

@Tubulin

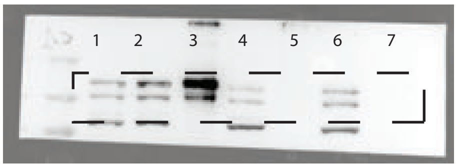

@SPAG5

FIGURE 5G

1-HCT116 WT Total
2- HCT116 WTmito crude
3 - HCT116WT mito pure

4-HCT116 CLUH KO Total
5- HCT116 CLUH KO mito crude
6 - HCT116 CLUH KO mito pure

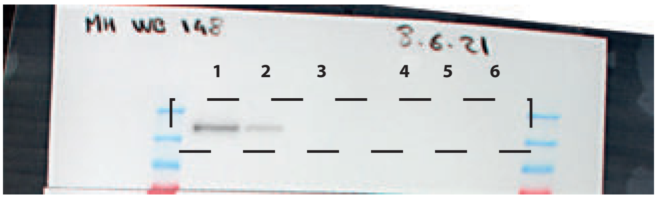

@CLUH

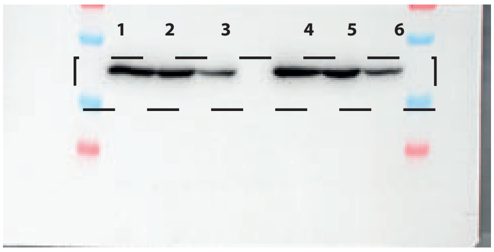

@Actin

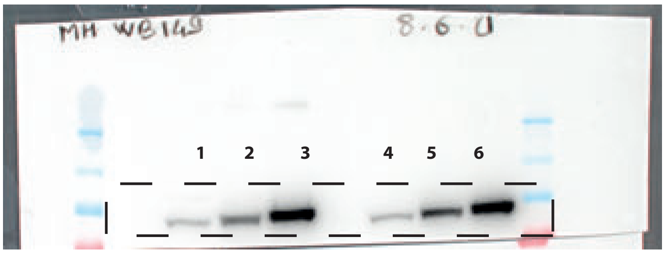

@IMMT

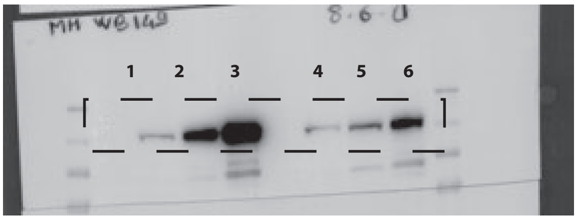

@LRPPRC

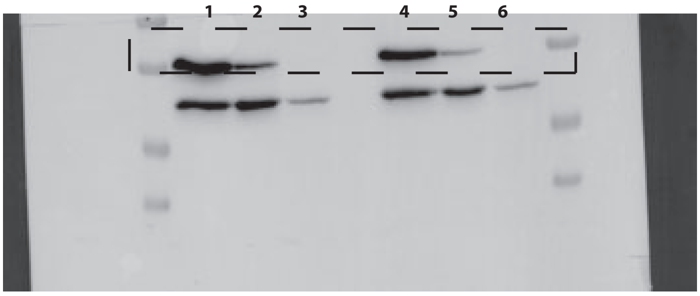

@TUBULIN

FIGURE 6E

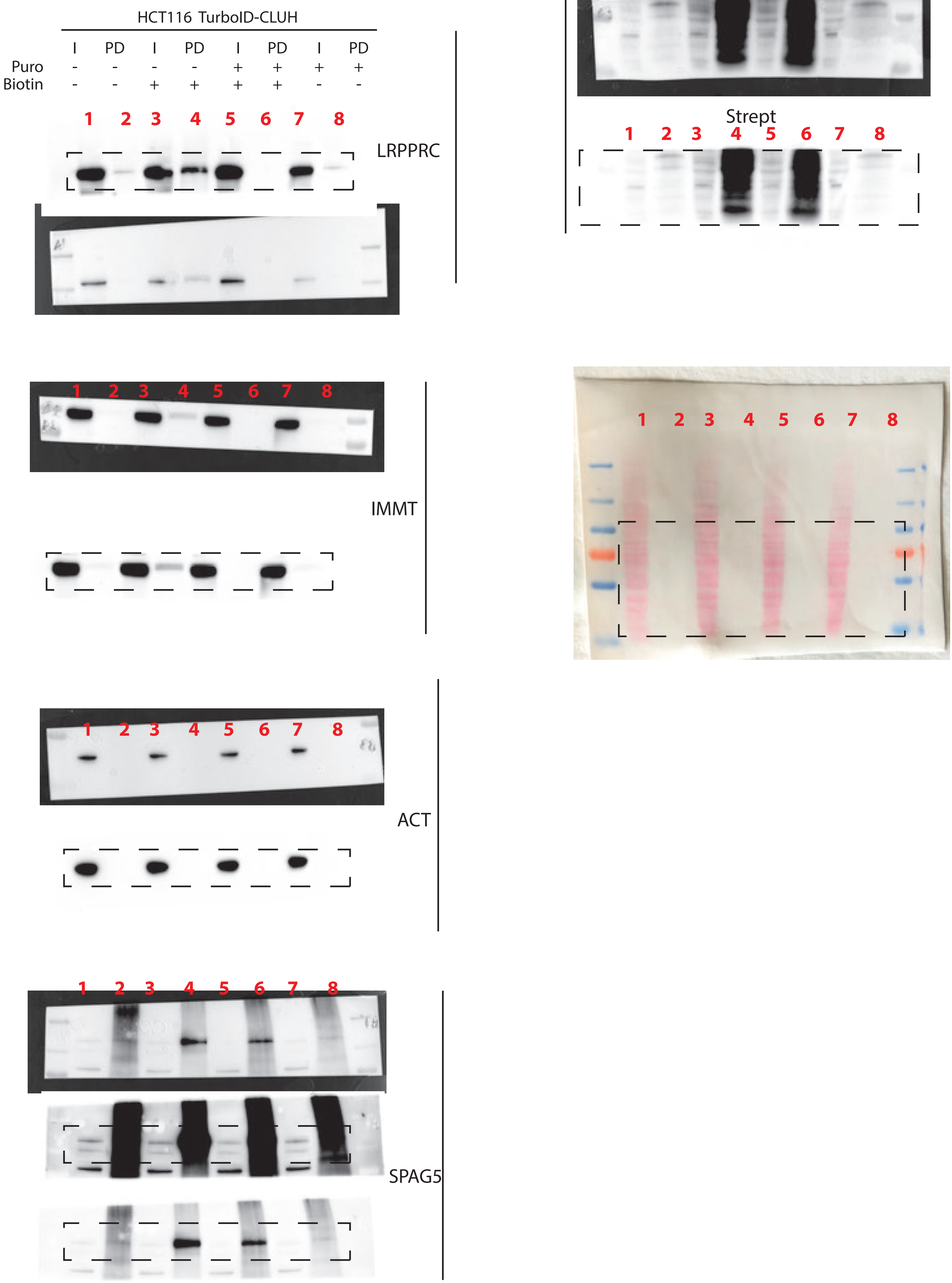
HCT116 TurboID-CLUH
I PD I PD I PD I PD
Puro - - - - + + + +
Biotin - - + + + + - -
1 2 3 4 5 6 7 8
LRPPRC
IMMT
ACT
SPAG5
Strept

FIGURE 7G

CLUH

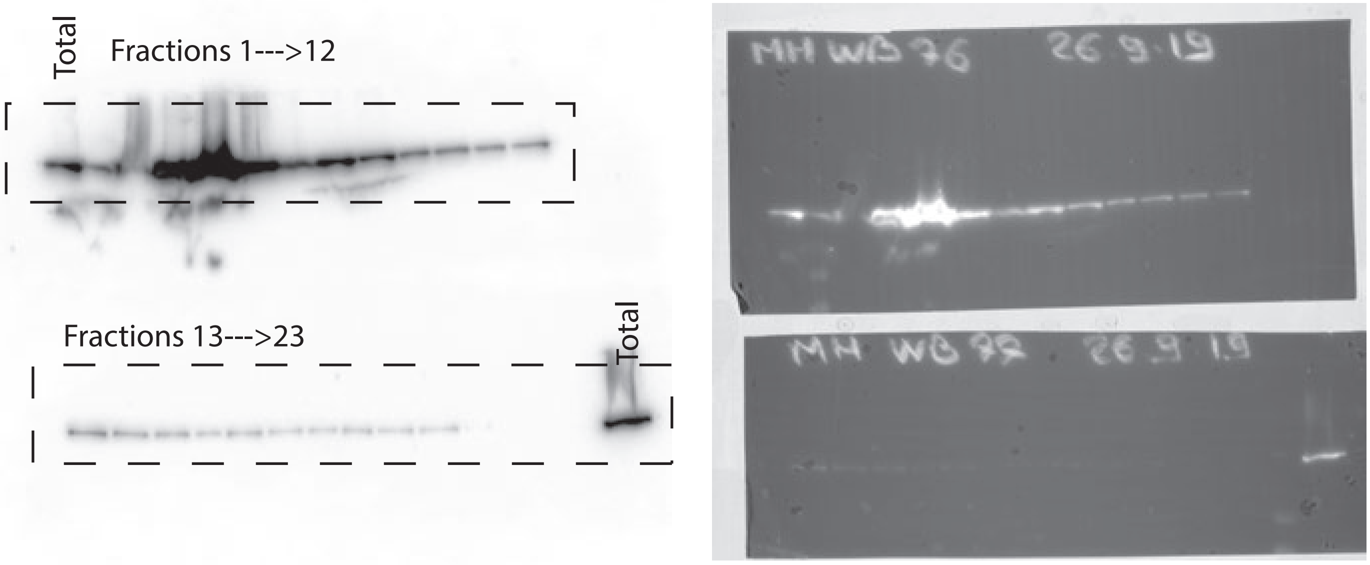

RPL7A

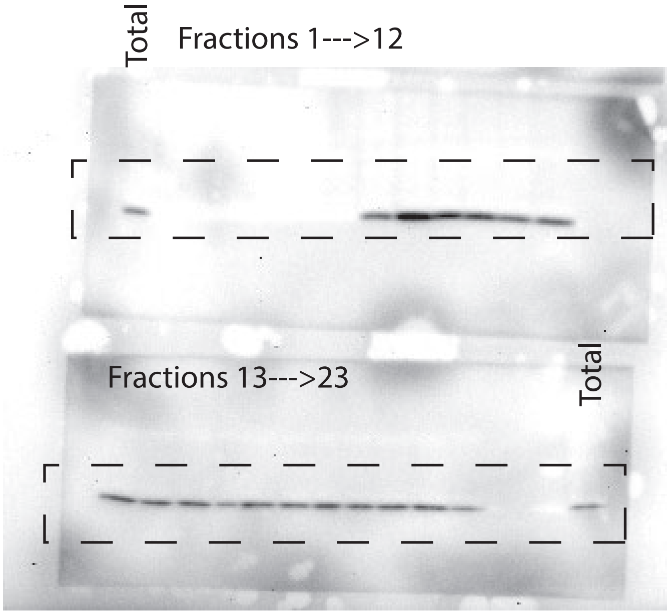

Tubulin

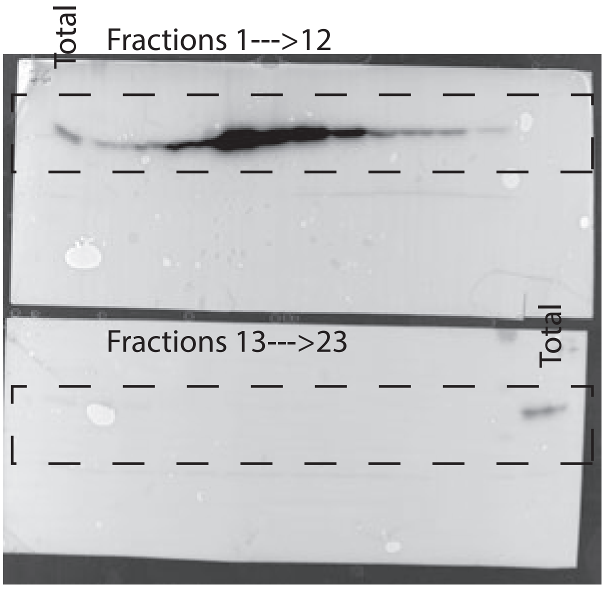

RPL14

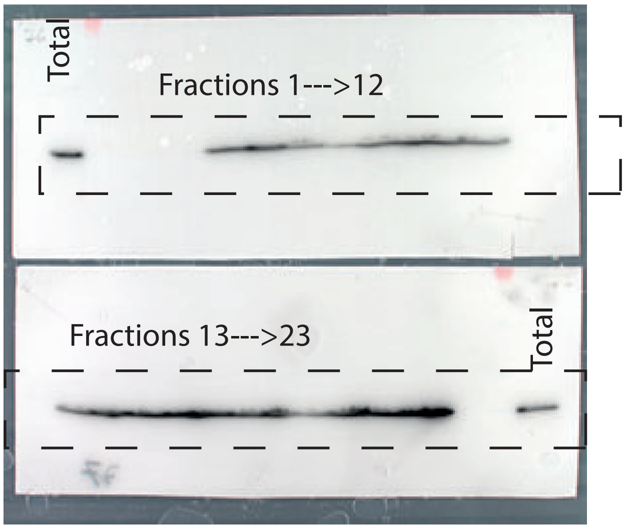

Figure S1A

1- HCT116 WT
2 - HCT116 - 3xHA-mCLUH

1 2

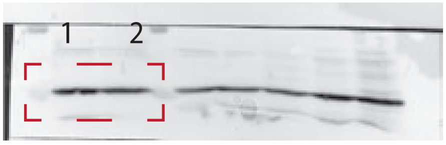

TUBULIN

1 2

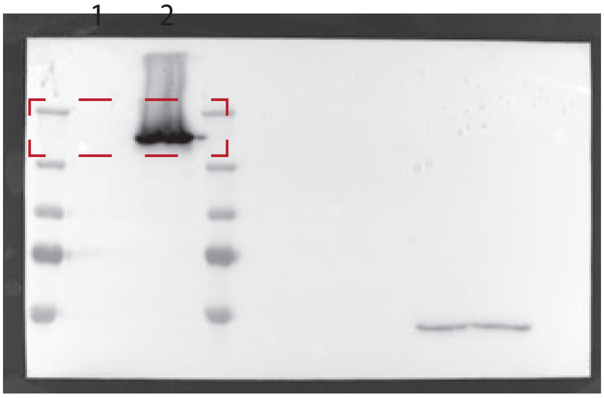

@HA

1 2

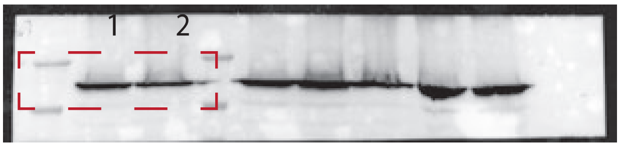

@CLUH (Novus)

Figure S1C

1 - E14 WT
2 - E14 KI 3xhA-CLUH G6
3 - E14 KI 3xhA-CLUH G12

1 2 3

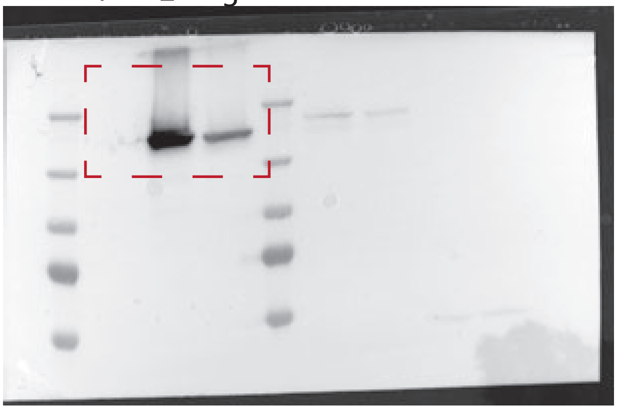

@HA

1 2 3

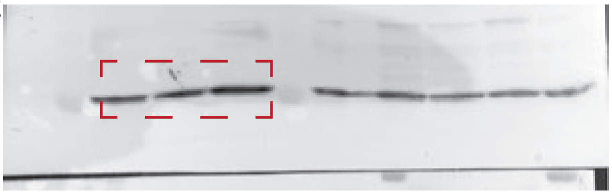

TUBULIN

1 2 3

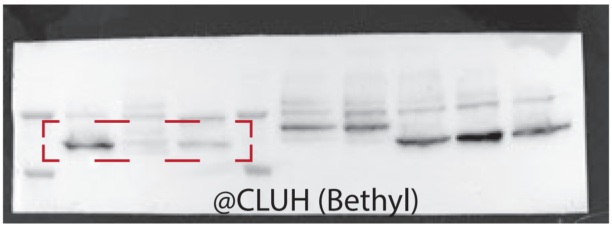

Figure S1D

1 - INPUT - HCT116 WT
2 - IP HA - HCT116 WT
3 - INPUT - HCT116 - 3xHA-CLUH
4- IP HA - HCT116 - 3xHA-CLUH
5 - INPUT - HCT116 - 3xHA-CLUH
6- IP HA - HCT116 - 3xHA-CLUH
7 - INPUT - HCT116 - 3xHA-CLUH
8- IP HA - HCT116 - 3xHA-CLUH

1 2 3 4 5 6 7 8

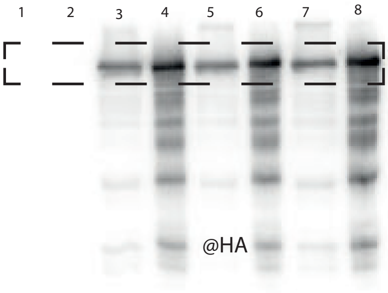

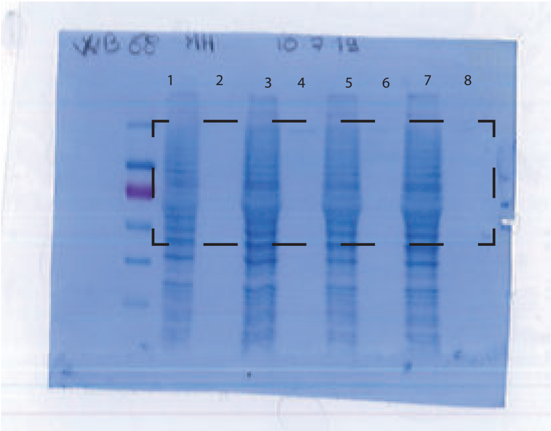

FIGURE S2A-B

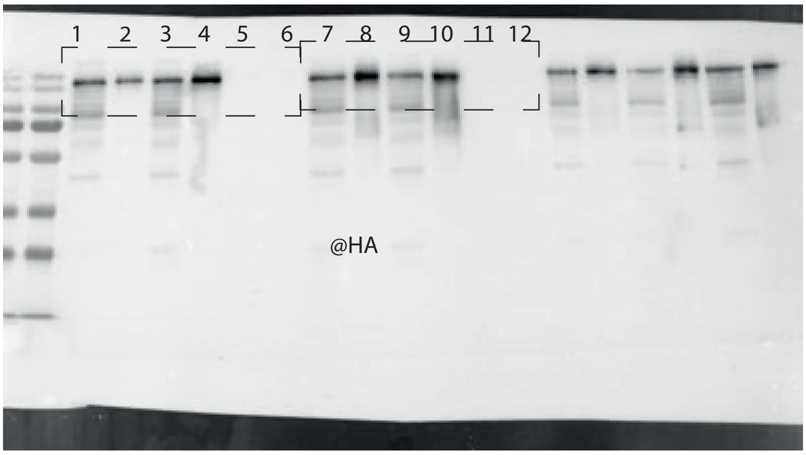

1 - Input HA-CLUH + GFP SPAG5
2- IP_HA HA-CLUH + GFP SPAG5
3 - Input HA-CLUH + GFP SPAG5 + RNAse
4- IP_HA HA-CLUH + GFP SPAG5 + RNAse
5 - Input GFP SPAG5
6- IP_HA GFP SPAG5
7 - Input HA-CLUH + GFP KNSTRN
8- IP_HA HA-CLUH + GFP KNSTRN
9 - Input HA-CLUH + GFP KNSTRN+ RNAse
10- IP_HA HA-CLUH + GFP KNSTRN+ RNAse
11 - Input GFP KNSTRN
12- IP_HA GFP KNSTRN

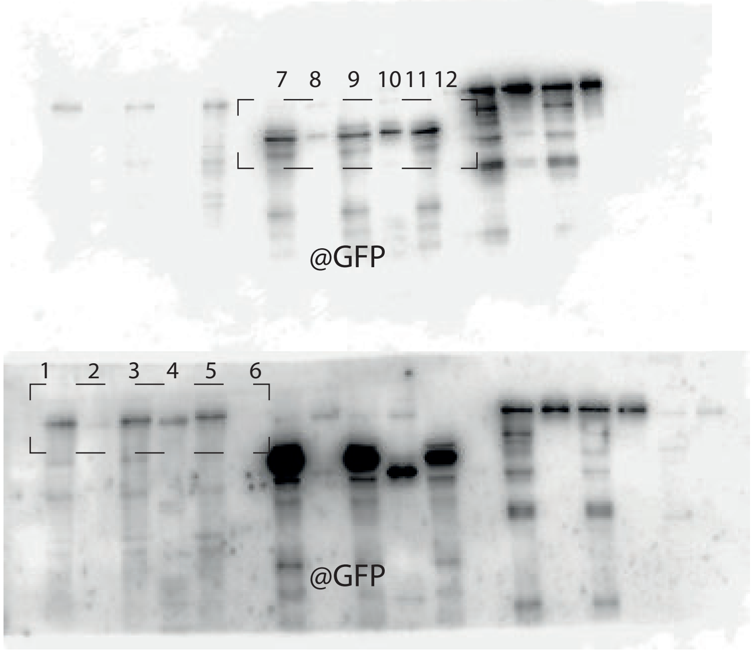

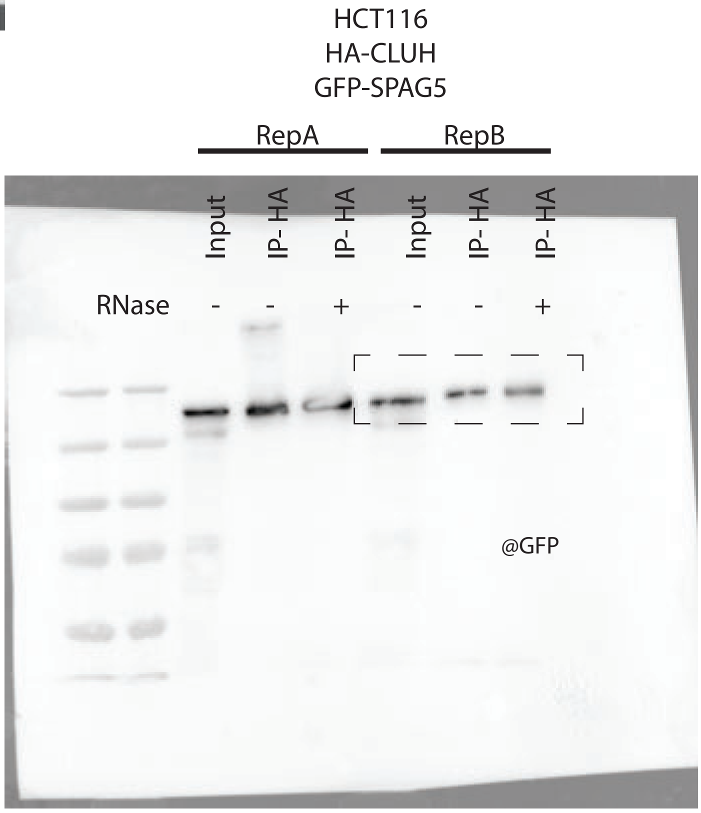

FIGURE S2C

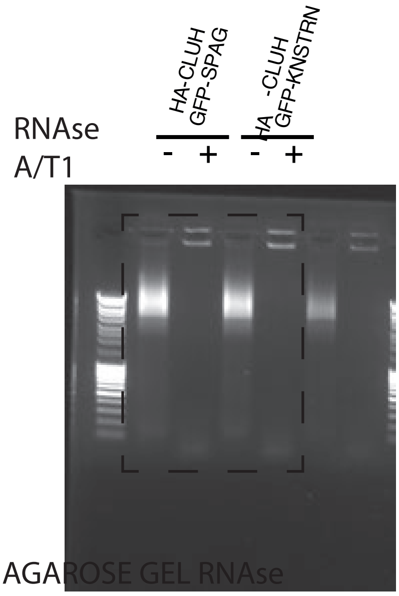

FIGURE S4

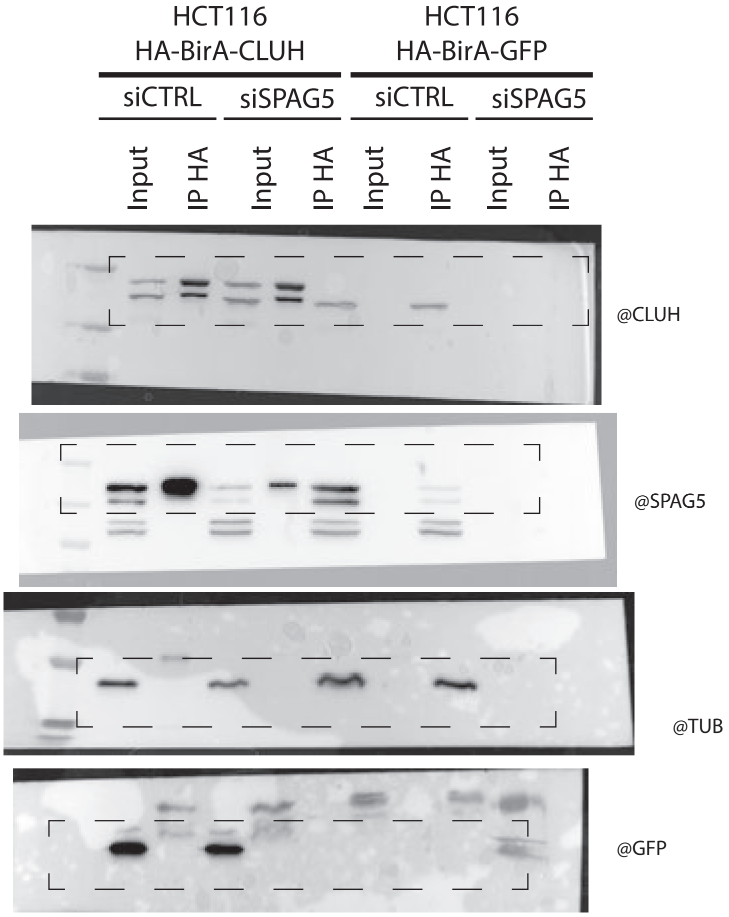
HCT116
HA-BirA-CLUH
HCT116
HA-BirA-GFP
siCTRL
siSPAG5
siCTRL
siSPAG5
Input
IP HA
Input
IP HA
Input
IP HA
Input
IP HA
@CLUH
@SPAG5
@TUB
@GFP

FIGURE S5A-B-D

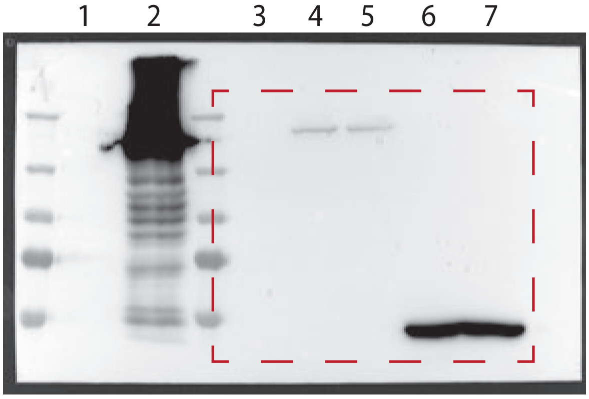

@HA

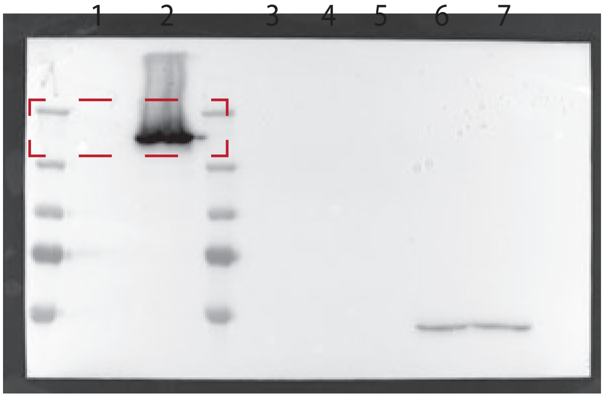

@HA

1- HCT116 WT
2 - HCT116 - 3xHA-mCLUH
3- HCT116 WT
4- HCT116 -BioID2-mCLUH
5 - HCT116-BioID2-mCLUH + Bio
4- HCT116 -BioID2-gfp
5 - HCT116-BioID2-gfp + Bio
Lorem ipsum

6 - E14 WT
7 - E14 KI 3xhA-CLUH G6
8 - E14 KI 3xhA-CLUH G12

9 - E14 KI BioID-CLUH E2 no Bio
10 - E14 KI BioID-CLUH E2 + Bio
11 - E14 BioID-gfp a2 no Bio
12 - E14 BioID-gfp a2 + Bio
13 - E14 WT

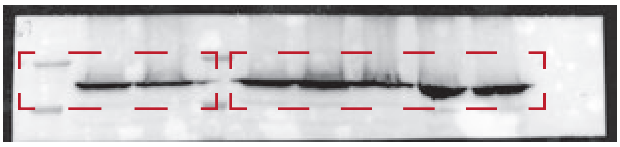

@CLUH (Novus)
Not recognizing mouse CLUH

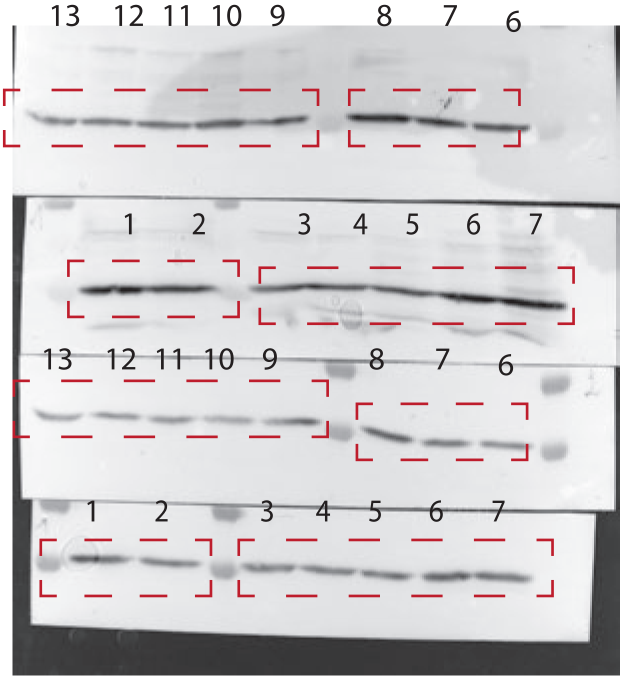

@Tubulin

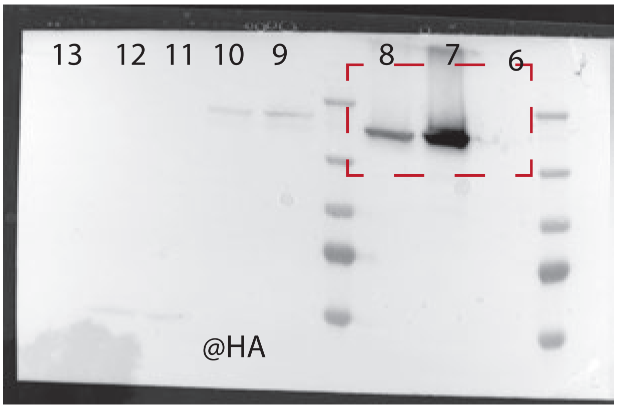

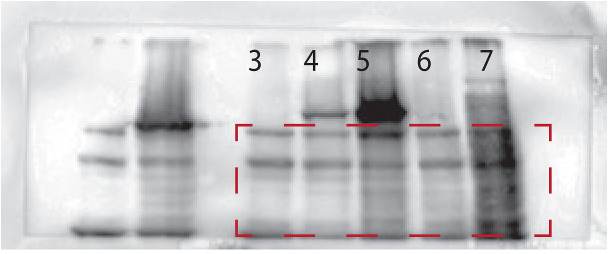

@strepta-HRP

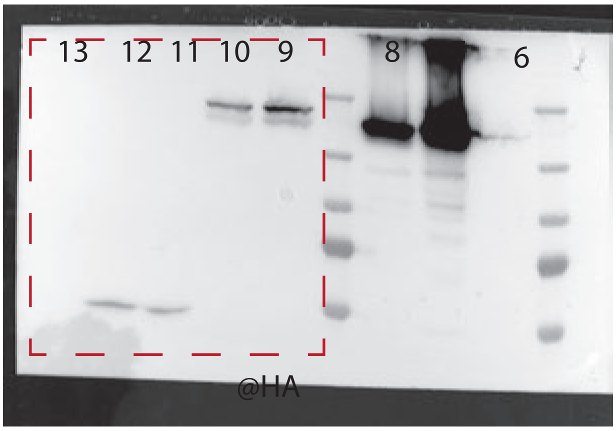

@HA

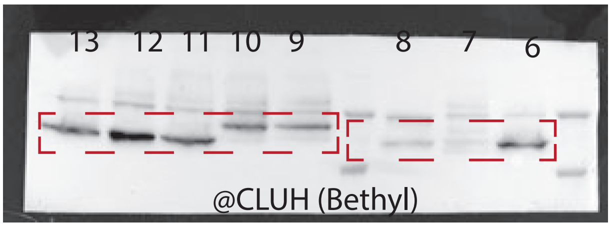

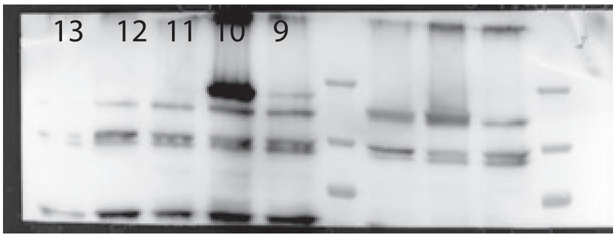

@strepta-HRP

Figure S6F

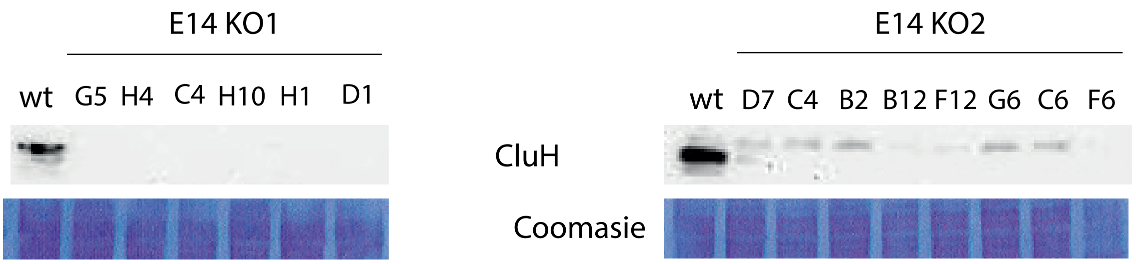

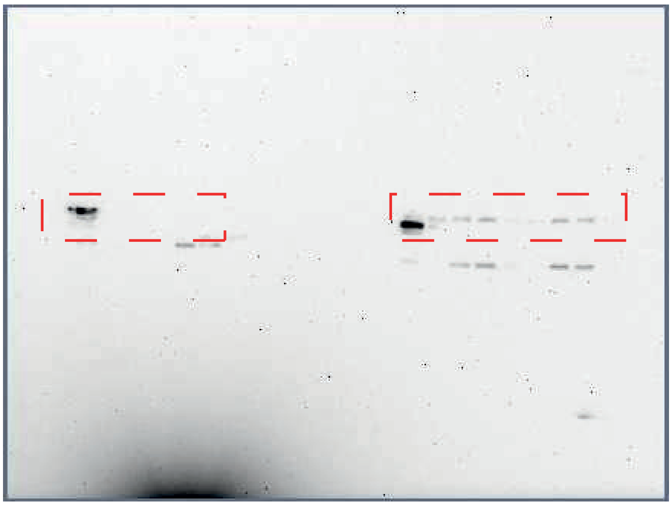

Figure S6J

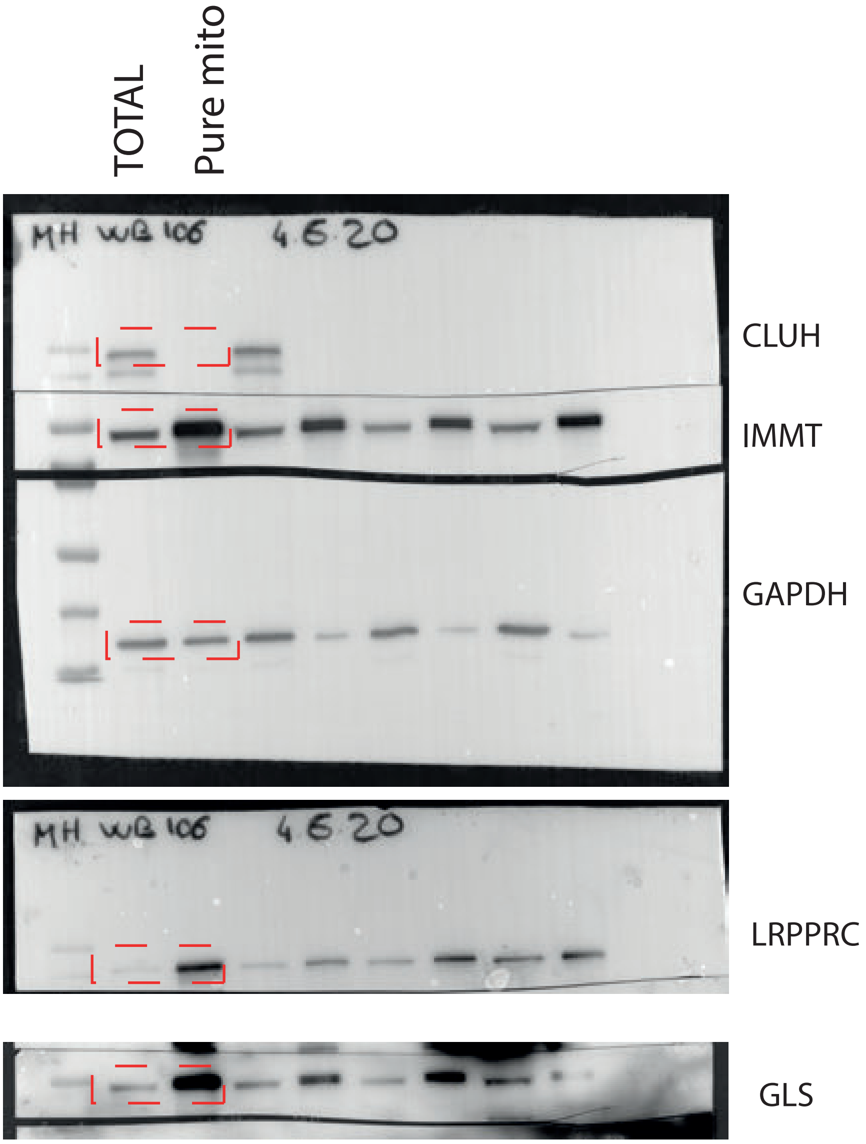

FIGURE S7B

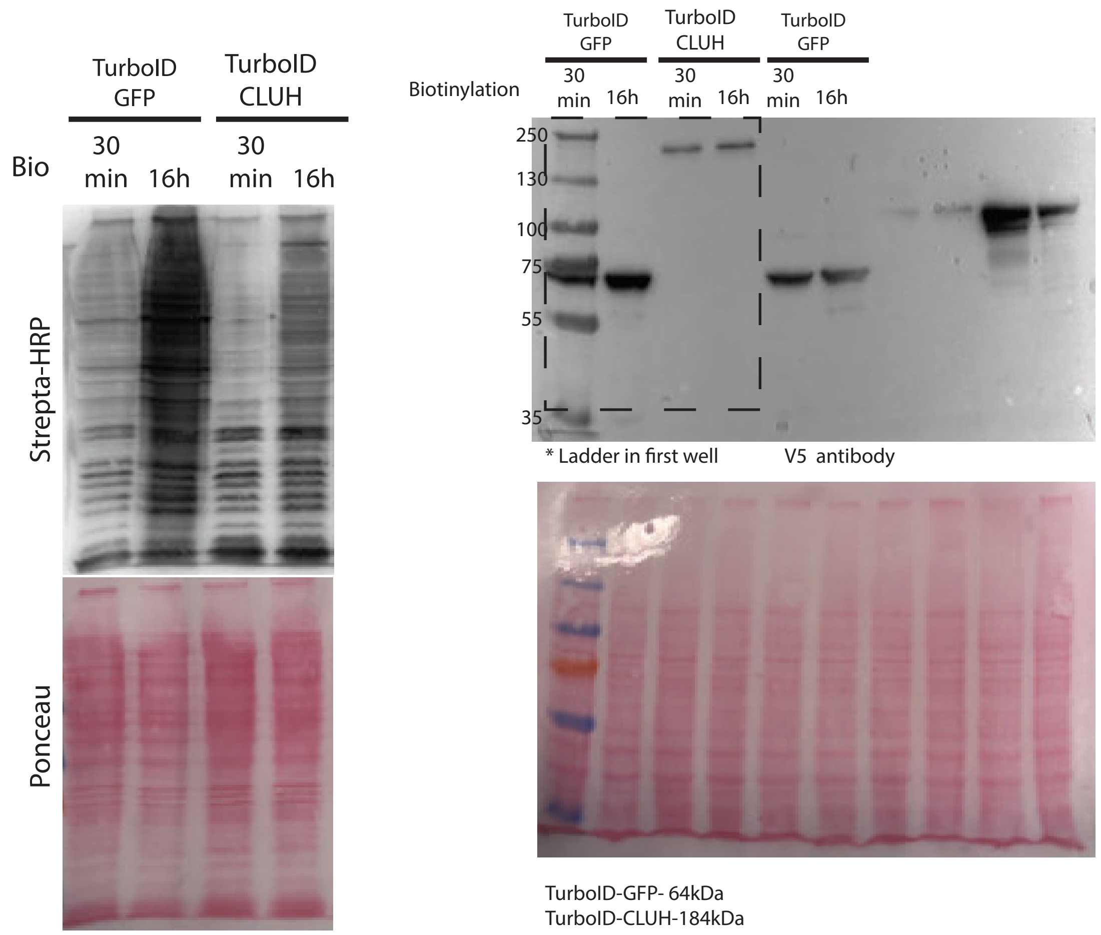

FIGURE S7H

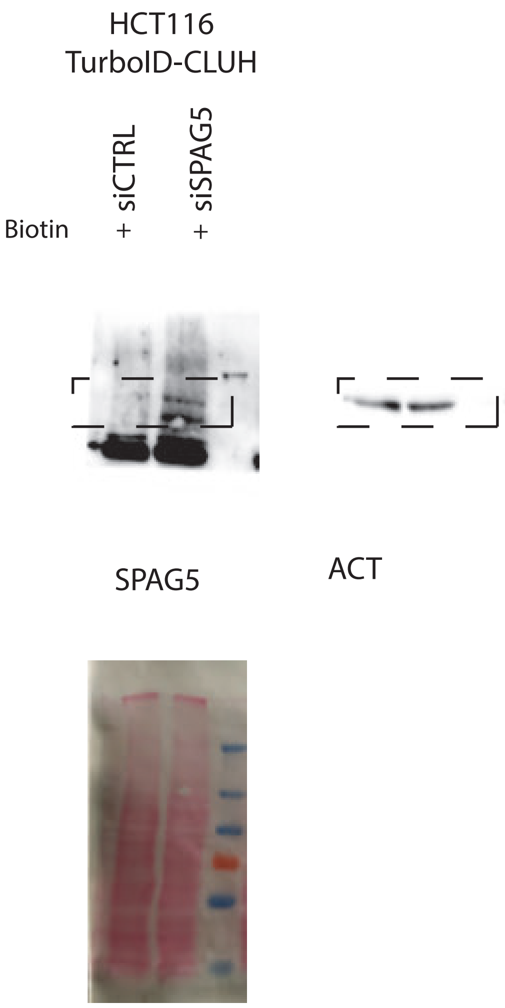

FIGURE S7H

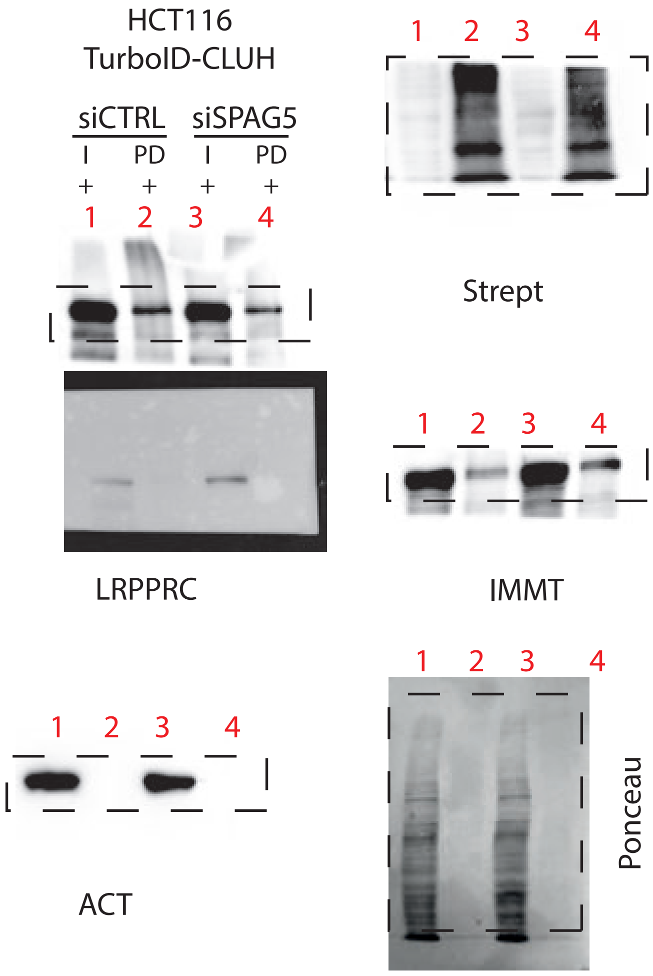

FIGURE S8D

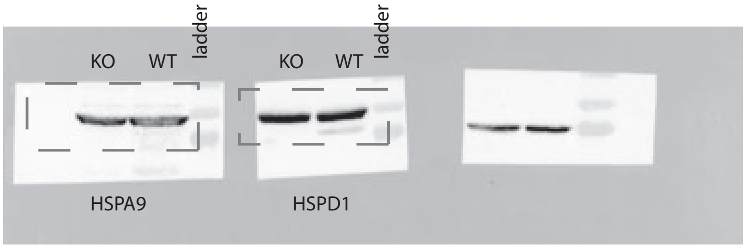

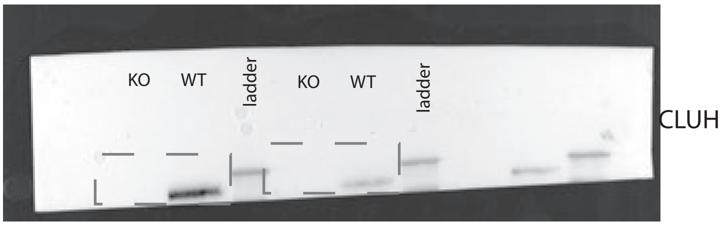

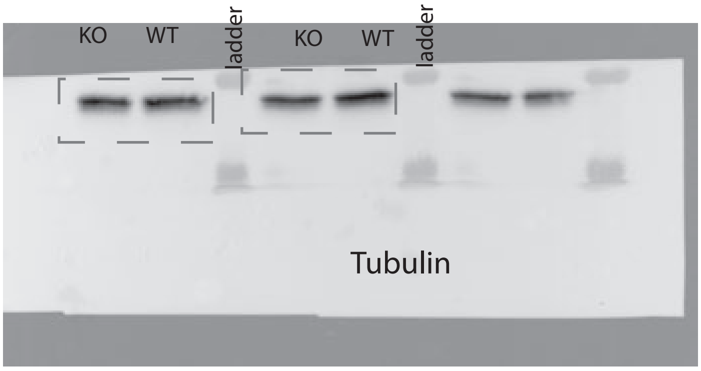

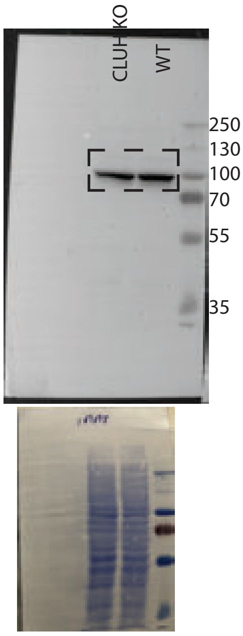

@IMMT
CSB PA623101LA01HU
Dillution 1:2000
84kDa

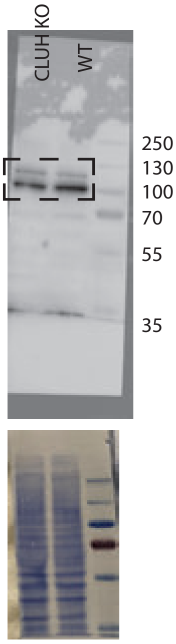

@IARS2
CSB-PA002995
Dillution 1:2000
114 kDa

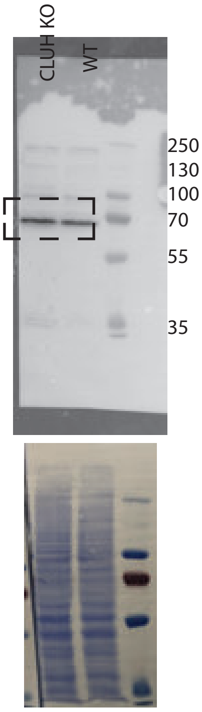

@GLS
E-AB-19340
Dillution 1:2000
73kDa

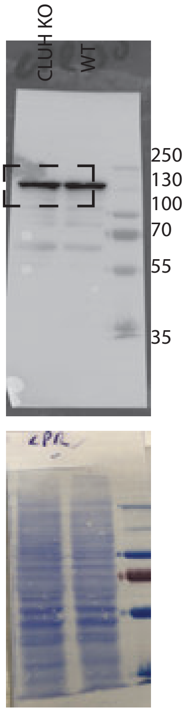

@LRP130 (LRPPRC)
GTX109558
Dillution 1:2000
158kDa obs.130 kDa

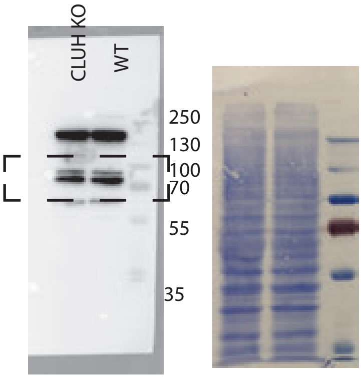

@NDUFS1
E-AB-11432
Dillution 1:2000
79 kDa

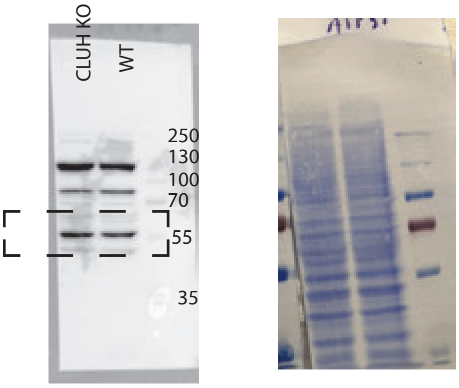

@ATP5A
E-AB-11432
Dillution 1:5000
60kDa

FIGURE S8F

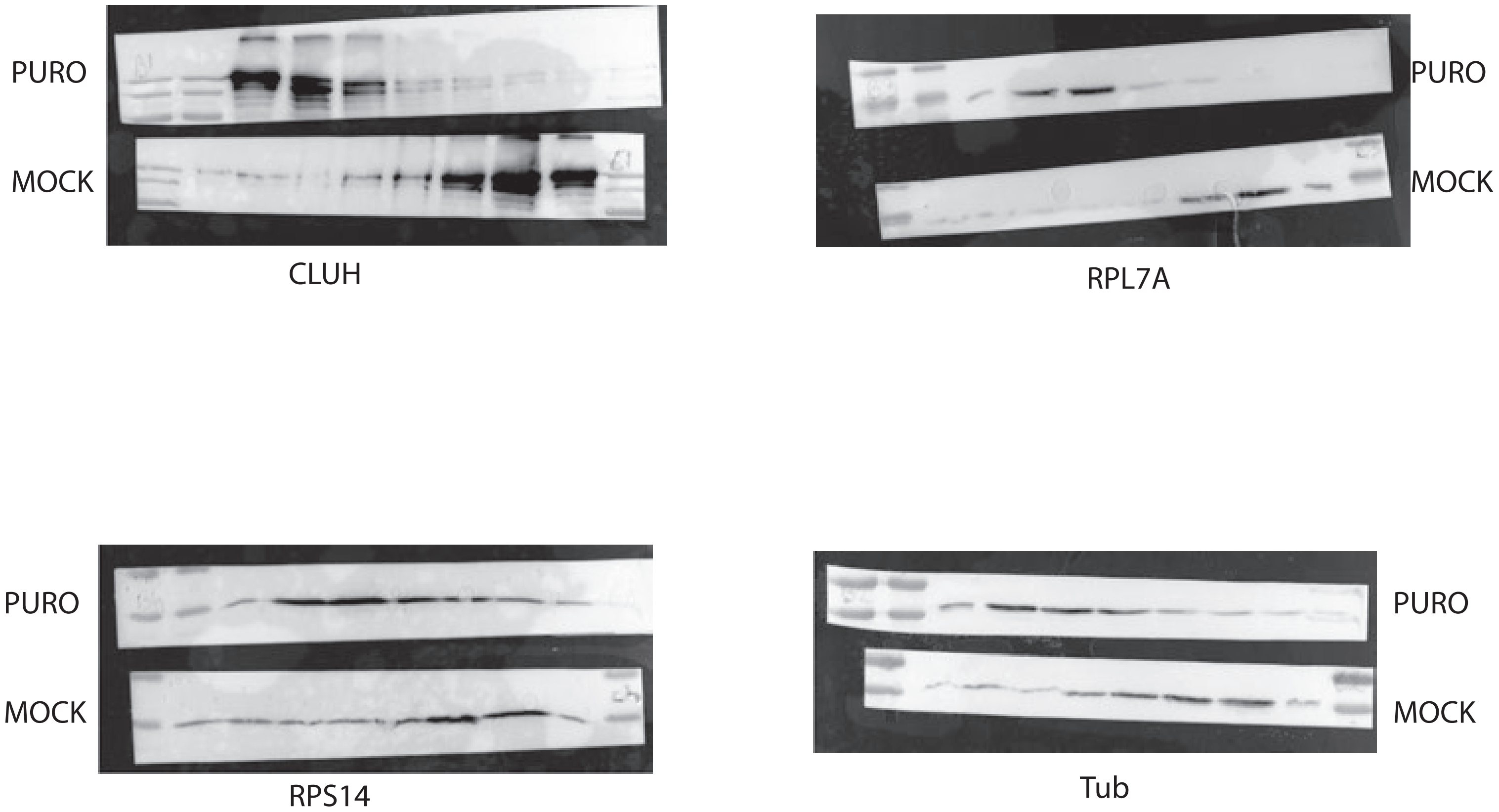

FIGURE S9B

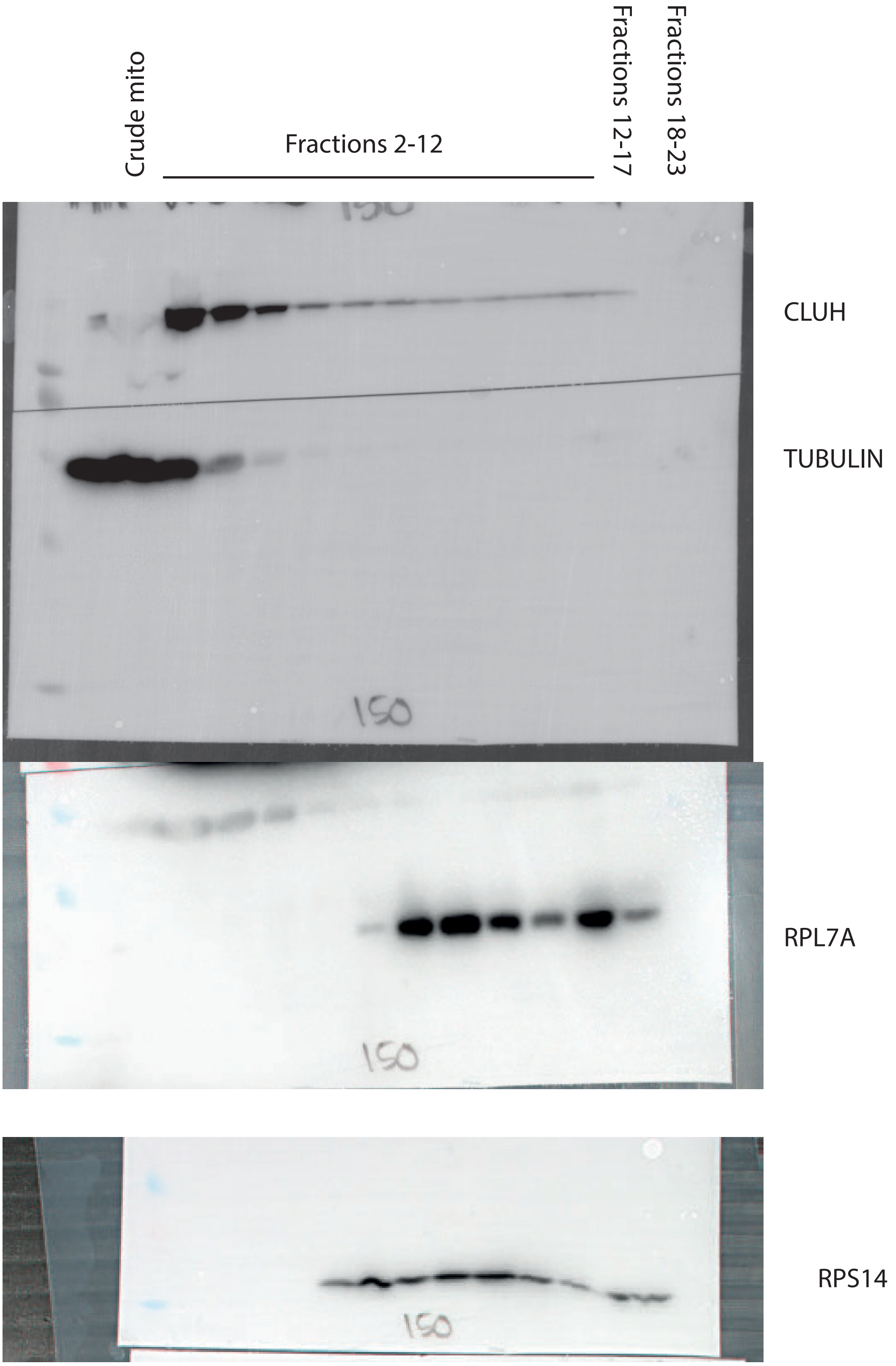

Crude mito
Fractions 2-12
Fractions 12-17
Fractions 18-23
CLUH
TUBULIN
RPL7A
RPS14
